# Patient‑specific profiling of TCR‑T cell efficacy enabled by a rapid and standardized reporter system

**DOI:** 10.1093/procel/pwag027

**Published:** 2026-03-26

**Authors:** Li Lu, Mengmeng Wei, Xiangxing Kong, Chenggang Hong, Wenhui Wei, Yulu Chen, Yuxin Zhu, Minghui He, Yushen Lin, Chaoqun Pan, Shuangshuang Liu, Jianqing Yu, Bo Yang, Zhan Zhou, Ji Cao, Wenbin Zhao

**Affiliations:** College of Pharmaceutical Science, Innovation Institute for Artificial Intelligence in Medicine of Zhejiang University, Zhejiang University, Hangzhou 310058, China; College of Pharmaceutical Science, Innovation Institute for Artificial Intelligence in Medicine of Zhejiang University, Zhejiang University, Hangzhou 310058, China; Department of Hepatobiliary and Pancreatic Surgery, The First Affiliated Hospital, Zhejiang University School of Medicine, Zhejiang University, Hangzhou 310058, China; Department of Colorectal Surgery and Oncology (Key Laboratory of Cancer Prevention and Intervention, China National Ministry of Education), The Second Affiliated Hospital, Zhejiang University School of Medicine, Zhejiang University, Hangzhou 310058, China; Center for Medical Research and Innovation in Digestive System Tumors, Ministry of Education, Hangzhou 310058, China; College of Pharmaceutical Science, Innovation Institute for Artificial Intelligence in Medicine of Zhejiang University, Zhejiang University, Hangzhou 310058, China; College of Pharmaceutical Science, Innovation Institute for Artificial Intelligence in Medicine of Zhejiang University, Zhejiang University, Hangzhou 310058, China; College of Pharmaceutical Science, Innovation Institute for Artificial Intelligence in Medicine of Zhejiang University, Zhejiang University, Hangzhou 310058, China; College of Pharmaceutical Science, Innovation Institute for Artificial Intelligence in Medicine of Zhejiang University, Zhejiang University, Hangzhou 310058, China; College of Pharmaceutical Science, Innovation Institute for Artificial Intelligence in Medicine of Zhejiang University, Zhejiang University, Hangzhou 310058, China; College of Pharmaceutical Science, Innovation Institute for Artificial Intelligence in Medicine of Zhejiang University, Zhejiang University, Hangzhou 310058, China; College of Pharmaceutical Science, Innovation Institute for Artificial Intelligence in Medicine of Zhejiang University, Zhejiang University, Hangzhou 310058, China; Zhejiang University School of Medicine, Zhejiang University, Hangzhou 310058, China; Department of Colorectal Surgery and Oncology (Key Laboratory of Cancer Prevention and Intervention, China National Ministry of Education), The Second Affiliated Hospital, Zhejiang University School of Medicine, Zhejiang University, Hangzhou 310058, China; College of Pharmaceutical Science, Innovation Institute for Artificial Intelligence in Medicine of Zhejiang University, Zhejiang University, Hangzhou 310058, China; School of Medicine, Hangzhou City University, Hangzhou 310015, China; College of Pharmaceutical Science, Innovation Institute for Artificial Intelligence in Medicine of Zhejiang University, Zhejiang University, Hangzhou 310058, China; State Key Laboratory of Advanced Drug Delivery and Release Systems, Zhejiang University, Hangzhou 310058, China; College of Pharmaceutical Science, Innovation Institute for Artificial Intelligence in Medicine of Zhejiang University, Zhejiang University, Hangzhou 310058, China; State Key Laboratory of Advanced Drug Delivery and Release Systems, Zhejiang University, Hangzhou 310058, China; Cancer Center, Zhejiang University, Hangzhou 310058, China; College of Pharmaceutical Science, Innovation Institute for Artificial Intelligence in Medicine of Zhejiang University, Zhejiang University, Hangzhou 310058, China

**Dear Editor,** 

T cell receptor-engineered T (TCR-T) cell therapy has recently moved from bench to bedside, as illustrated by the Food and Drug Administration (FDA) approval of MAGE-A4–targeted TCR-T cell therapy for advanced synovial sarcoma (Barnett et al., 2025). However, as the pivotal molecule for tumor recognition and T-cell activation, the peptide–human leukocyte antigen (pHLA) complex can be suppressed by tumor cells to drive immune evasion and compromise therapeutic efficacy of TCR-T cells (Nagarsheth et al., 2021). This occurs via diverse mechanisms, ranging from protein-level alterations, such as altered protein stability and proteasome remodeling, to genetic and transcriptional defects, including the downregulation of antigen-presentation gene expression and the disruption of HLA genetic integrity (Dersh et al., 2021). Current methods for assessing patient suitability for TCR-T cell therapy rely on genomic and transcriptomic sequencing to evaluate antigen expression and human leukocyte antigen (HLA) typing, coupled with bioinformatic pipelines that incorporate multiple parameters to predict candidate pHLAs (Baulu et al., 2023). However, these time‑intensive approaches neither fully recapitulate physiological antigen presentation nor provide direct evidence for the abundance of pHLAs or authentic T-cell responsiveness.

Jurkat‑based reporter systems offer a rapid assay platform for evaluating TCR‑T cell therapeutic potential in clinical samples by sensing early TCR activation triggered by antigen recognition (Abraham and Weiss, 2004). However, existing reporter systems vary substantially in design, sensitivity, and signal output, and the extent to which their readouts are functionally relevant to TCR‑T cell cytotoxic activity remains insufficiently characterized. Here, we establish a standardized Jurkat‑based reporter system and demonstrate that its readout robustly captures TCR‑T cell functionality *in vitro* and *in vivo*. Leveraging this platform, we further enable rapid assessment of TCR‑T cell therapeutic potential from patient‑derived *ex vivo* tissues, providing a functional basis for patient-specific stratification within 12 h.

Concretely speaking, a standardized TCR signaling-based functional assessment system (TCR-SignalFunc system) was established in Jurkat cells (clone E6-1) by knocking out endogenous TCR α and β constant regions (*TRAC/TRBC*) and incorporating a synthetic promoter containing eight tandem NFAT: AP-1 elements (8×NFAT: AP-1) for high-sensitivity activation sensing (Figs. 1A, 1B and S1A–F) (Seo et al., 2021). Given the digital response pattern of T-cell signaling, the SignalFunc index (percentage of ZsGreen-positive cells) was employed as the quantitative readout (Fig. S1G). This robust system can display detectable signaling within 3 h and requires as few as 400 target cells for activation, remaining unaffected by assay variability or excess non-target cells (Figs. 1C, 1D, and S1H–I). Furthermore, CD8 was incorporated into the system to better approximate the functional context of therapeutic TCR‑T cells. Consistent with CD8‑mediated stabilization of TCR–pHLA interactions (Qin et al., 2025), expression of the CD8 coreceptor enhanced the SignalFunc index in an affinity‑dependent manner, with the most pronounced effect observed for low‑affinity TCRs (Fig. 1E and 1F).

**Figure 1.**
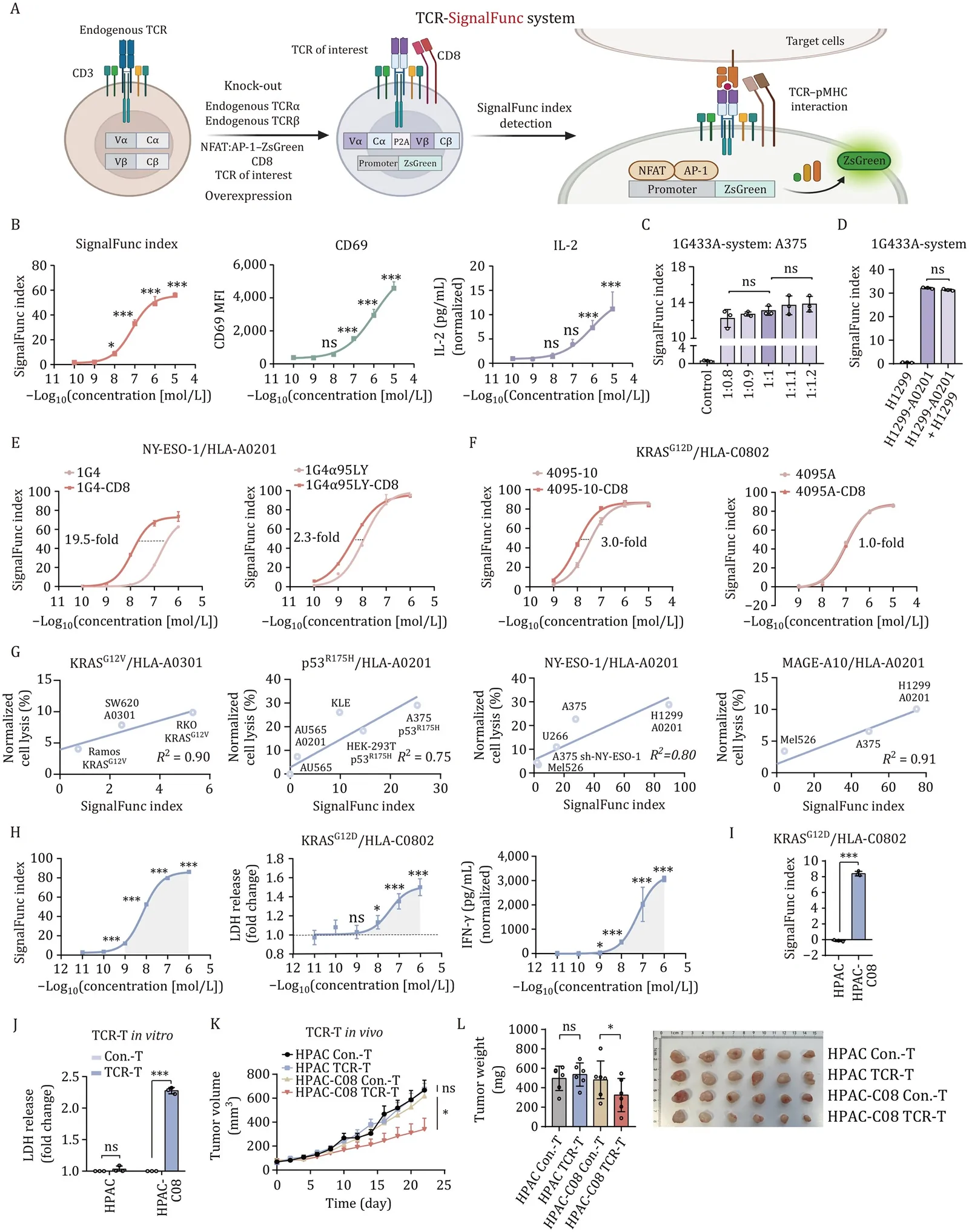
The robust TCR-SignalFunc system precisely reflects TCR-T cell functionality *in vitro* and *in vivo*. (A) Schematic of the TCR-SignalFunc system. Endogenous TCR-deficient reporter cells are transduced with CD8 and specific TCRs to convert TCR activation into ZsGreen fluorescence via the NFAT: AP-1 transcription factor complex. (B) Performance comparison with conventional activation markers. The TCR-SignalFunc system transduced with the 1G433A TCR specific for NY-ESO-1157–165/HLA-A0201 (1G433A-system) was co-cultured with peptide-pulsed T2 cells at an effector-to-target (E:T) ratio of 1:1 for 24 h. ZsGreen-positive cell ratio (SignalFunc index) and CD69 mean fluorescence intensity were measured by flow cytometry; IL-2 levels were determined by enzyme-linked immunosorbent assay (ELISA) and normalized. *P*-values were calculated relative to the 0 M peptide control. (C) Impact of cell ratios on assay potency and sensitivity. The 1G433A-system was co-cultured with A375 cells (NY-ESO-1^+^/HLA-A0201^+^) at varying E: T ratios for 24 h. (D) Assessment of non-target cell interference. The 1G433A-system was co-cultured with target cells (H1299-A0201) at a 1:1 ratio in the presence or absence of a 10-fold excess of non-target cells (H1299). (E–F) CD8 co-receptor modulation of the SignalFunc index. Systems with or without CD8 were co-cultured with tumor cells at graded peptide concentrations (E:T = 1:1, 24 h); fold changes represent shifts in EC_50_ values. Comparisons were performed between low- and high-affinity TCRs targeting (E) NY-ESO-1_157–165_/HLA-A0201 (1G4 *vs*. 1G4α95LY) using HEK-293T cells, and (F) KRAS^G12D^/HLA-C0802 (4095-10 *vs*. 4095A) using K562-C08 cells. (G) Correlation between TCR-T cell cytotoxicity and the SignalFunc index. Normalized cell lysis (Annexin V^+^ proportion) and the SignalFunc index were compared using identical TCRs. (H) Dose–response sensitivity of the SignalFunc index versus TCR-T cell activity. The 4095-10-system (targeting KRAS^G12D^/HLA-C0802) and corresponding TCR-T cells were co-cultured with K562-C08 cells at graded peptide concentrations (E:T = 1:1, 24 h). Readouts comprised the SignalFunc index, normalized IFN-γ levels (determined by ELISA), and LDH release. *p*-values were calculated relative to the 0 M peptide control. (I–J) Functional association in the HPAC tumor model. Assessment of the 4095-10-system (I) and TCR-T cells (J) against HPAC (KRAS^G12D+^/low HLA-C0802) and HPAC-C08 cells (HLA-C0802-overexpressing) (E:T = 1:1, 24 h). (K–L) The anti-tumor activity of 4095-10 TCR-T cells in HPAC or HPAC-C08 xenografts. Mice received 4095-10 TCR-T or control-T cells via tail vein injection (4.48 × 10^6^ cells/mouse, *n* = 6). (K) Tumor volume curves and (L) tumor weights at day 22 post-infusion. Data are presented as mean ± SEM in (K) and as mean ± SD in all other panels (ns, *P *≥ 0.05; *P < 0.05; **P < 0.01; ***P < 0.001).

We next evaluated the performance of the TCR‑SignalFunc system across diverse antigen targets and assessed whether the SignalFunc index could robustly predict TCR‑T cell function. For each TCR under evaluation, the corresponding SignalFunc system was constructed as a Jurkat reporter cell line expressing only this single TCR. Using TCR‑SignalFunc systems transduced with TCRs specific to KRAS^G12V^/HLA-A0301 (TCRA3V TCR), p53^R175H^/HLA-A0201 (4196-AV12/BV6 TCR), NY-ESO-1_157–165_/HLA-A0201 (1G4α95LY TCR), and MAGE-A10_254-262_/HLA-A0201 (c796 TCR), the resulting SignalFunc index correlated strongly with the *in vitro* cytotoxicity of the corresponding TCR‑T cells (*R*^2^ ≥ 0.75) (Figs. 1G and S2A–F). Moreover, the TCR-SignalFunc system effectively distinguished immune responsiveness between HLA-C0802-engineered and parental HPAC cells (KRAS G12D^+^, low HLA-C0802 expression), as stimulation with HPAC-C08 cells elicited a robust response, whereas parental cells did not (Fig. 1I). This activation profile mirrored the behavior of 4095-10 TCR-T cells, which selectively lysed HPAC-C08 but not parental HPAC cells in both *in vitro* and *in vivo* models (Figs. 1H, 1J–L and S3A–C). These findings demonstrate that the TCR-SignalFunc system effectively recapitulates TCR-T cell responsiveness and serves as a reliable surrogate for predicting anti-tumor efficacy.

Subsequently, we sought to apply the system to *ex vivo* patient-derived tissue samples to achieve rapid evaluation of TCR-T cell therapeutic potential for cancer patients. Compared with passaged cell lines, processing *ex vivo* tissue samples necessitate two additional steps: dissociation into a single-cell suspension and estimation of the tumor-cell proportion. We first verified that a 30-minute treatment with tissue dissociation solution did not compromise surface pHLA expression in passaged cell lines (Fig. S4A). A375 cell line-derived xenografts (CDXs) were then dissociated, and the tumor-cell proportion was quantified via surface HLA-A02 staining. Upon co-culture with the NY-ESO-1–specific TCR-SignalFunc system, the SignalFunc index from CDX suspensions was comparable to that of passaged A375 cells, demonstrating the system’s robustness against enzymatic digestion and non-target-cell admixture (Fig. S4B and S4C).

Since tumor and non-tumor cells in patient tissues share the same HLA subtype, HLA-based estimation of tumor proportion was not feasible in clinical samples. Given that alterations in DNA content, including polyploidy and hypoploidy, are well-recognized hallmarks of cancer (Cornish et al., 2024), DNA ploidy was evaluated as a surrogate (Georvasili et al., 2022). In MC38 CDXs engineered to express enhanced green fluorescent protein (EGFP), propidium iodide (PI)-based DNA content analysis yielded an aneuploid proportion (PI positive) that closely matched the proportion of EGFP positive cells, supporting the use of aneuploidy as an approximate quantitative estimate of tumor cell proportion in tissue suspensions (Fig. S4D and S4E).

Building on these results, we developed a clinical workflow to assess TCR-T cell therapeutic potential directly from patient-derived tissues. HLA genotyping was performed on peripheral blood using flow cytometry and high-resolution sequencing. Surgically resected tumor and adjacent non-tumorous tissues were dissociated into single-cell suspensions, and the tumor-cell proportion was estimated via DNA-ploidy analysis. By co-culturing a standardized number of these cells with TCR-transduced SignalFunc systems, we utilized the SignalFunc index as a functional readout to quantify patient-specific therapeutic responsiveness (Fig. 2A).

**Figure 2.**
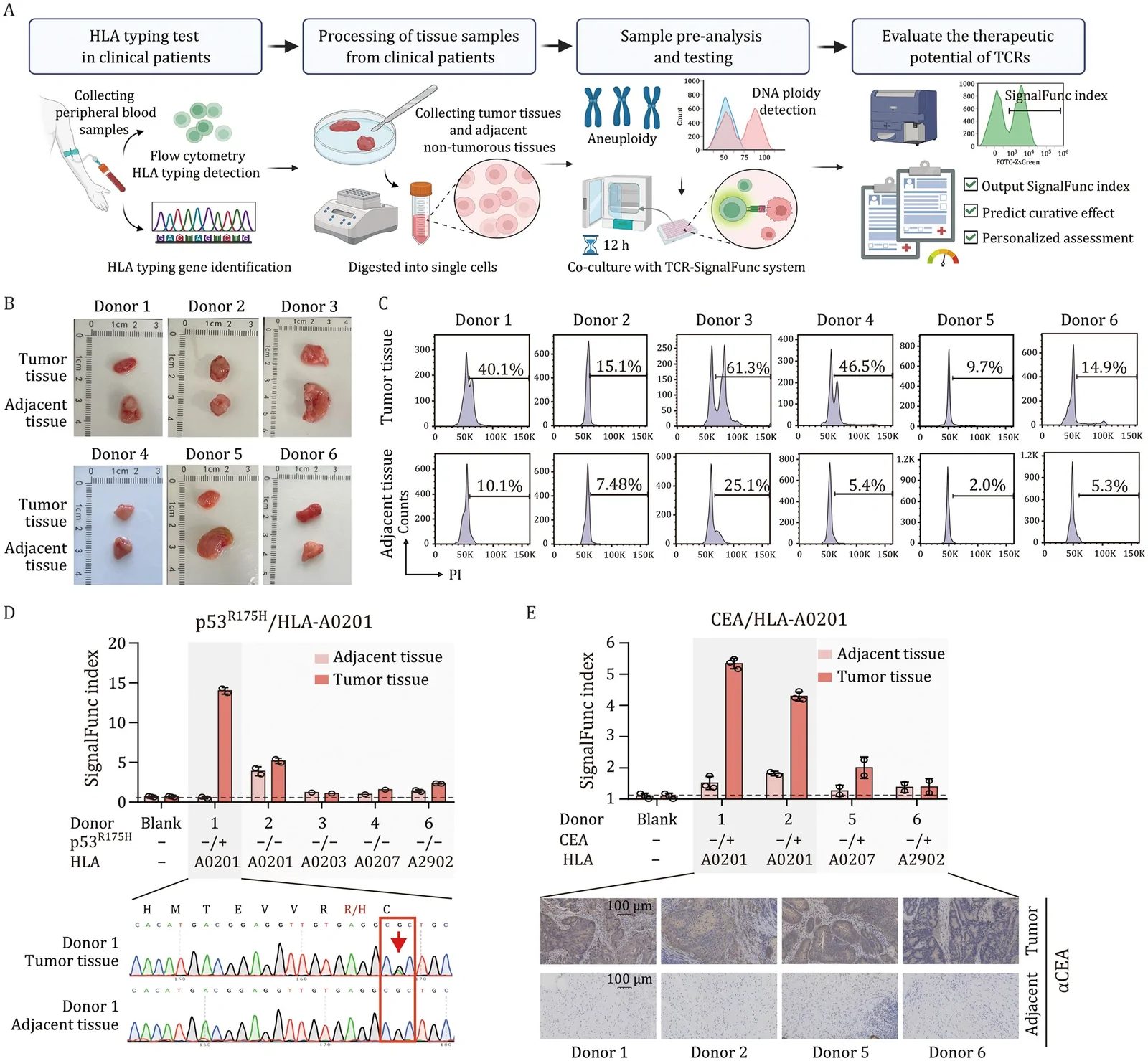
Rapid assessment of TCR-T cell therapeutic potential in patient-derived samples via the TCR-SignalFunc system. (A) Workflow for patient-specific TCR-T therapeutic evaluation. Peripheral blood was collected to isolate peripheral blood mononuclear cells, and HLA subtypes were determined by flow cytometry and genetic analysis. Tumor and adjacent non-tumorous tissues were digested into single-cell suspensions, and the proportion of tumor cells was estimated by DNA ploidy analysis. Single-cell suspensions were co-cultured with the relevant SignalFunc system (E:T = 1:1, 12 h), and the SignalFunc index was measured to determine patient-specific TCR-T therapeutic potential. (B) Photos of patient-derived colorectal tumor and adjacent non-tumorous tissues obtained via surgical resection. (C) DNA ploidy analysis of single-cell suspensions using propidium iodide (PI) staining. Cells were incubated for 10 minutes in PBS containing 0.25 μL/mL Triton X-100, 0.05 mg/mL RNase, and 0.05 mg/mL PI, followed by flow cytometric analysis. (D) Evaluation of the therapeutic potential of 4196-AV12/BV6 TCR-T (targeting p53^R175H^/HLA-A0201). The engineered system was co-cultured with single-cell suspensions of tumor tissues and adjacent non-tumorous tissues from different donors (E:T = 1:1, 12 h). The p53 R175H mutation was confirmed by gene sequencing. (E) Evaluation of the therapeutic potential of CEA TCR-T (targeting CEA_691–699_/HLA-A0201). The engineered system was co-cultured with single-cell suspensions of tumor tissues and adjacent non-tumorous tissues from different donors (E:T = 1:1, 12 h). CEA expression was confirmed by immunohistochemistry. All replicates are shown as mean ± SD.

As a prevalent marker for colorectal cancer, carcinoembryonic antigen (CEA) is presented as the CEA_691–699_/HLA-A0201 complex, offering a promising TCR-T cell therapy target for treating colorectal cancer (Parkhurst et al., 2011). In addition, the p53 R175H mutation occurs at high frequency in colorectal cancers, and its corresponding neoantigen p53^R175H^/HLA-A0201 exhibits strong tumor specificity (Lo et al., 2019). Accordingly, we collected tumor and adjacent non-tumorous tissues from six colorectal cancer donors to assess the therapeutic potential of TCRs recognizing p53^R175H^/HLA-A0201 (4196-AV12/BV6 TCR) and CEA_691–699_/HLA-A0201 (CEA TCR) (Figs. 2B, 2C, and S5A–B). The 4196-AV12/BV6-system exhibited a robust, specific response to tumor tissue from donor 1, who harbored p53 R175H mutation and expressed HLA-A0201, whereas no response was detected in tumors from donors 2, 3, 4, and 6 lacking this mutation (Figs. 2D and S5C). Similarly, the CEA TCR-system responded specifically to tumors expressing both CEA and HLA-A0201, but not to CEA-positive, HLA-A0201-negative tumor tissues (Fig. 2E). These results demonstrate that the TCR-SignalFunc system enables direct *ex vivo* measurement of TCR-T cell function and provides a practical framework for predicting patient-specific TCR-T cell efficacy.

Beyond pHLA downregulation, tumors evade immune surveillance by modulating co-stimulatory and co-inhibitory pathways to dampen T‑cell activation (Fig. S6A). Loss of CD58, which disrupts the CD2–CD58 axis, has been linked to immune escape and resistance to immune checkpoint blockade in melanoma and to chimeric antigen receptor T‑cell (CAR‑T) therapy in lymphoma (Ho et al., 2023). Consistent with this, antibody‑mediated CD58 blockade in the TCR‑SignalFunc system reduced the SignalFunc index (Fig. S6B). Similarly, introduction of programmed cell death protein 1 (PD-1) and evaluation of programmed death-ligand 1 (PD-L1)–high cell lines also resulted in SignalFunc index suppression (Fig. S6C) (Lin et al., 2024). Together, these findings indicate that the TCR‑SignalFunc system, enabled by multiplexed editing, holds potential for precise prediction of TCR‑T cell therapeutic efficacy in a more comprehensive context.

The TCR-SignalFunc system offers several practical advantages for clinical application. Robust reporter readouts can be achieved using a minimal number of tumor cells (greater than 400), supporting its compatibility with the evaluation of limited clinical specimens, including small-volume tissue samples such as endoscopic biopsies obtained without surgical intervention. In addition, the system enables rapid, high‑throughput functional assessment within 12 h, providing timely guidance for preliminary decision‑making regarding patient eligibility for TCR‑T cell therapy. The system is also highly programmable, allowing integration of additional signaling inputs to further improve predictive accuracy. Overall, this system provides a promising alternative strategy for patient selection in TCR‑T cell therapy. Although clinical limitations in this study prevented the infusion of TCR‑T cell products into the sample donors and thus precluded direct validation of the readouts against treatment outcomes, the system nonetheless establishes a practical framework for evaluating TCR‑T cell therapeutic potential directly in patient‑derived tissue specimens. Notably, the use of dissociated patient‑derived cells in two‑dimensional monocultures may not fully reflect the intricate complexity of the native tumor microenvironment. Accordingly, future integration of the TCR‑SignalFunc system with more physiologically relevant models, such as tumor organoid cultures that preserve tissue architecture and cell–cell interactions (van de Wetering et al., 2015), could further refine its predictive accuracy. In addition, future multiplexing of SignalFunc systems using distinct fluorescently labeled TCRs could enable simultaneous evaluation of multiple TCR activities in a single pooled assay, substantially improving screening throughput and efficiency.

The interpretation of the SignalFunc index is central to the application of this system. The SignalFunc index enables quantitative comparison of responses for the same TCR under matched conditions, but absolute values should be interpreted with caution across different TCRs when expression levels or experimental conditions differ. A SignalFunc index that is significantly elevated above background is likely to indicate detectable functional activation of the corresponding TCR‑T cells, with higher values generally reflecting greater functional potency. However, because TCR‑T activity is also influenced by factors such as TCR expression levels, co‑stimulatory, or co‑inhibitory signals, defining a universal activation threshold applicable across all contexts remains challenging. Furthermore, establishing a clinically ­actionable threshold will necessitate future studies that correlate SignalFunc index measurements from patient‑derived samples with clinical response metrics, such as objective response rate, duration of response, and survival outcomes. Importantly, given the potential for false‑negative results, whether low or non‑responsive SignalFunc index values should be definitively interpreted as indicating clinical non‑responsiveness warrants further investigation. Given the high cost, prolonged treatment course, and variable efficacy of TCR‑T cell therapy, prioritizing robust positive selection for further evaluation aligns with clinical risk management and resource optimization, underscoring the practical value of this platform.

Given the strong correlation between TCR‑SignalFunc system readouts and TCR‑T therapeutic efficacy observed both *in vitro* and *in vivo*, we further explored the application of this platform for preliminary assessment of the cross‑reactivity and optimization of TCRs. We evaluated the specificity of clinically tested TCRs (Fig. S7A–C) as well as the cross-reactivity of additional TCRs with differ­ing affinities targeting KRAS^G12D^/HLA-A11 (Fig. S7D–F). Furthermore, library-based parallel screening within the SignalFunc system enabled high-throughput optimization of the KRAS^G12D^/HLA-A11-specific JDI TCR, identifying enhanced candidates such as the CDR3α-S11H mutant (Fig. S8A–I). Together, these exploratory studies demonstrate that the TCR‑SignalFunc system represents a robust and versatile platform with broad applicability for TCR therapeutic evaluation and optimization.

Overall, we present a standardized and robust reporter system that reliably recapitulates TCR‑T cell cytotoxic activity both *in vitro* and *in vivo*. By enabling rapid, patient-specific assessment of therapeutic potential from *ex vivo* tissue samples within 12 h, this platform provides a functional readout with substantial translational utility for early patient stratification and therapeutic evaluation in TCR‑T cell therapy.

## Supplementary data

Supplementary data is available at *Protein & Cell* Journal online https://doi.org/10.1093/procel/pwag027.

## Footnotes

This work was supported by the National Natural Science Foundation of China (Grant Nos. 82473827, Wen-bin Zhao; 82303384, Jian-qing Yu; U25D9023, Bo Yang; China); the Fundamental Research Funds for the Central Universities (Grant Nos. 226-2025-00009, Wen-bin Zhao, China); the National Key Research and Development Program of China (Grant Nos. 2022YFA1305800, Zhan Zhou, China); and the Zhejiang Province “Pioneer and Leading Goose + X” Science and Technology Program (Grant Nos. 2024C03167, Zhan Zhou, China). Figures for this article are created with BioRender.com.

All animal maintenance and experimental procedures for mouse studies were approved by the Institutional Animal Care and Use Committee (IACUC) of the Innovation Institute for Artificial Intelligence in Medicine, Zhejiang University (DW202408151333). Surgically resected tissue specimens were collected from patients at the Second Affiliated Hospital, Zhejiang University School of Medicine (China) with written informed consent and in accordance with the guidelines of the hospital’s Human Research Ethics Committee (approval no. 2022-LYS-0959).

The authors declare no conflict of interest.

The datasets generated during and/or analysed during the current study are available from the corresponding author on reasonable request.

Conceptualization (Li Lu, Meng-meng Wei and Xiang-xing Kong), Methodology (Li Lu, Meng-meng Wei, Xiang-xing Kong, Cheng-gang Hong, Wen-hui Wei, Yu-lu Chen, Yu-xin Zhu, Ming-hui He, Yu-shen Lin, Chao-qun Pan, Shuang-shuang Liu, Bo Yang, Zhan Zhou, Ji Cao and Wen-bin Zhao); Experimental work (Li Lu, Meng-meng Wei, Xiang-xing Kong, Cheng-gang Hong, Wen-hui Wei, Yu-lu Chen, Yu-xin Zhu, Ming-hui He, Yu-shen Lin, Chao-qun Pan); Data analysis (Li Lu, Meng-meng Wei, Xiang-xing Kong, Cheng-gang Hong, Wen-hui Wei, Yu-lu Chen, Yu-xin Zhu, Ming-hui He, Yu-shen Lin, Chao-qun Pan, Shuang-shuang Liu, Bo Yang, Zhan Zhou, Ji Cao and Wen-bin Zhao); Visualization (Li Lu, Meng-meng Wei, Xiang-xing Kong, Cheng-gang Hong, Wen-hui Wei, Yu-lu Chen, Yu-xin Zhu, Ming-hui He, Yu-shen Lin, Chao-qun Pan, Shuang-shuang Liu, Bo Yang, Zhan Zhou, Ji Cao and Wen-bin Zhao), Funding acquisition (Wen-bin Zhao, Ji Cao, Bo Yang, Jian-qing Yu and Zhan Zhou), Project administration (Wen-bin Zhao, Ji Cao, Zhan Zhou and Bo Yang), Supervision (Wen-bin Zhao, Ji Cao, Zhan Zhou and Bo Yang), Writing—original draft (Li Lu); Writing—review & editing (all authors).

We used AI-assisted technologies (ChatGPT, OpenAI) solely for language polishing and grammar improvement of the manuscript. All scientific content, analyses, and conclusions were conceived, written, and verified by the authors.

## Supplementary Material

pwag027_Supplementary_Data

## References

[pwag027-B1] Abraham RT , Weiss A. Jurkat T cells and development of the T-cell receptor signalling paradigm. Nat Rev Immunol 2004;4:301–308. 10.1038/nri1330 15057788

[pwag027-B2] Barnett KK , Johnson AR, Das A et al FDA approval summary: Afamitresgene autoleucel for adults with HLA-Restricted, MAGE-A4-Positive unresectable or metastatic synovial sarcoma after prior chemotherapy. Clin Cancer Res 2025;31:3112–3117. 10.1158/1078-0432.CCR-25-0595 PMC12316539 40423661

[pwag027-B3] Baulu E , Gardet C, Chuvin N et al TCR-engineered T cell therapy in solid tumors: state of the art and perspectives. Sci Adv 2023;9:eadf3700. 10.1126/sciadv.adf3700 PMC9931212 36791198

[pwag027-B4] Cornish AJ , Gruber AJ, Kinnersley B et al The genomic landscape of 2,023 colorectal cancers. Nature 2024;633:127–136. 10.1038/s41586-024-07747-9 PMC11374690 39112709

[pwag027-B5] Dersh D , Holly J, Yewdell JW. A few good peptides: MHC class I-based cancer immunosurveillance and immunoevasion. Nat Rev Immunol 2021;21:116–128. 10.1038/s41577-020-0390-6 32820267

[pwag027-B6] Georvasili VK , Markopoulos GS, Batistatou A et al Detection of cancer cells and tumor margins during colorectal cancer surgery by intraoperative flow cytometry. Int J Surg 2022;104:106717. 10.1016/j.ijsu.2022.106717 35724804

[pwag027-B7] Ho P , Melms JC, Rogava M et al The CD58-CD2 axis is co-regulated with PD-L1 via CMTM6 and shapes anti-tumor immunity. Cancer Cell 2023;41:1207–1221 e1212. 10.1016/j.ccell.2023.05.014 PMC10524902 37327789

[pwag027-B8] Lin X , Kang K, Chen P et al Regulatory mechanisms of PD-1/PD-L1 in cancers. Mol Cancer 2024;23:108. 10.1186/s12943-024-02023-w PMC11102195 38762484

[pwag027-B9] Lo W , Parkhurst M, Robbins PF et al Immunologic recognition of a shared p53 mutated neoantigen in a patient with metastatic colorectal cancer. Cancer Immunol Res 2019;7:534–543. 10.1158/2326-6066.CIR-18-0686 PMC6685528 30709841

[pwag027-B10] Nagarsheth NB , Norberg SM, Sinkoe AL et al TCR-engineered T cells targeting E7 for patients with metastatic HPV-associated epithelial cancers. Nat Med 2021;27:419–425. 10.1038/s41591-020-01225-1 PMC9620481 33558725

[pwag027-B11] Parkhurst MR , Yang JC, Langan RC et al T cells targeting carcinoembryonic antigen can mediate regression of metastatic colorectal cancer but induce severe transient colitis. Mol Ther 2011;19:620–626. 10.1038/mt.2010.272 PMC3048186 21157437

[pwag027-B12] Qin R , Zhang Y, Shi J et al TCR catch bonds nonlinearly control CD8 cooperation to shape T cell specificity. Cell Res 2025;35:265–283. 10.1038/s41422-025-01077-9 PMC11958657 40011760

[pwag027-B13] Seo H , Gonzalez-Avalos E, Zhang W et al BATF and IRF4 cooperate to counter exhaustion in tumor-infiltrating CAR T cells. Nat Immunol 2021;22:983–995. 10.1038/s41590-021-00964-8 PMC8319109 34282330

[pwag027-B14] van de Wetering M , Francies HE, Francis JM et al Prospective derivation of a living organoid biobank of colorectal cancer patients. Cell 2015;161:933–945. 10.1016/j.cell.2015.03.053 PMC6428276 25957691

