# Supplementary material for: Patient‑specific profiling of TCR‑T cell efficacy enabled by a rapid and standardized reporter system
Source: Protein Cell. 2026 Mar 26;17(10):981–6. doi: 10.1093/procel/pwag027 (PMC13626734; doi:10.1093/procel/pwag027)
Supplement: Supplementary file 1 [file pwag027_supplementary_data.pdf]

Supporting Information for

Patient-Specific Profiling of TCR-T Cell Efficacy Enabled by a Rapid and Standardized Reporter System

Li Lu[1#], Meng-meng Wei[1,2#], Xiang-xing Kong[3,4#], Cheng-gang Hong[1], Wen-hui Wei[1], Yu-lu Chen[1], Yu-xin Zhu[1], Ming-hui He[1], Yu-shen Lin[1], Chao-qun Pan[1], Shuang-shuang Liu[5], Jian-qing Yu[3], Bo Yang[1,6], Zhan Zhou[1,7*], Ji Cao[1,7,8*], Wen-bin Zhao[1*]*

1. College of Pharmaceutical Science, Innovation Institute for Artificial Intelligence in Medicine of Zhejiang University, Zhejiang University, Yuhangtang Road 866, Hangzhou 310058, Zhejiang, China.
2. Department of Hepatobiliary and Pancreatic Surgery, the First Affiliated Hospital, Zhejiang University School of Medicine, Hangzhou, China.
3. Department of Colorectal Surgery and Oncology (Key Laboratory of Cancer Prevention and Intervention, China National Ministry of Education), The Second Affiliated Hospital, Zhejiang University School of Medicine, Hangzhou, China.
4. Center for Medical Research and Innovation in Digestive System Tumors, Ministry of Education, Hangzhou, China.
5. Zhejiang University School of Medicine, Hangzhou, China.
6. School of Medicine, Hangzhou City University, Hangzhou, China.
7. State Key Laboratory of Advanced Drug Delivery and Release Systems, Zhejiang University, Hangzhou, China.
8. Cancer Center, Zhejiang University, Hangzhou, China.

# Equal contribution.
* Corresponding authors. Tel: 86-571-882028401, Fax: 86-571-88208400.
E-mail addresses: pharmacy_zwb@zju.edu.cn (Wen-bin Zhao); caoji88@zju.edu.cn (Ji Cao); zhanzhou@zju.edu.cn (Zhan Zhou); yang924@zju.edu.cn (Bo Yang).

This PDF file includes:

Materials and Methods

Figures S1 to S8

Tables S1 to S2

Materials and Methods

Ethical Approval

All animal maintenance and experimental procedures for mouse studies were approved by the Institutional Animal Care and Use Committee (IACUC) of the Innovation Institute for Artificial Intelligence in Medicine, Zhejiang University (DW202408151333). Surgically resected tissue specimens were collected from patients at the Second Affiliated Hospital, Zhejiang University School of Medicine (China) with written informed consent and in accordance with the guidelines of the hospital's Human Research Ethics Committee (approval no. 2022-LYS-0959).

TCRs and target antigens

TCRs used in this study were either generated in-house or obtained from published sources. All TCRs were characterized for their cognate peptide epitopes and restricting HLA alleles. Detailed information, including TCR ID, antigen source, epitope sequence, epitope position, and corresponding HLA restriction, is provided in **Table S1**.

Cell lines

HEK-293T, A375, Mel526, LS174T, MDA-MB-231, MDA-MB-436, MDA-MB-157 cells were cultured in DMEM (Gibco) with 10% fetal bovine serum (FBS; Hyclone). Jurkat, K562, T47D, CHO-K1, MC38, U266, Raji, Ramos, AU565, H1299, COLO 205, Mino, H1975, BT-549 and their stable cell lines were cultured in RPMI-1640 (Gibco) with 10% FBS. Stably transfected T47D cells required the addition of 10 μg/mL insulin to the medium. HCT116 and their stable cell lines were cultured in McCoy's 5A (Gibco) with 10% FBS. KLE was cultured in DMEM/F12 (Gibco) with 10% FBS. SW480, SW620 and their stable cell lines were cultured in Leibovitz's L-15 (Gibco) with 10% FBS. T2, MV4-11 were cultured in IMDM (Gibco) with 10% FBS. RKO was cultured in MEM (Gibco) with 10% FBS. HPAC and their stable cell lines were cultured in HPAC cell complete medium (Procell). PBMCs and their stable cell lines were cultured in X-VIVO15 (Lonza) with 10% FBS (Gibco), 100 U/mL penicillin and 100 μg/mL streptomycin. All cell lines used in this study were authenticated by short tandem repeat (STR) profiling to confirm their origin and identity.

Furthermore, mycoplasma contamination was tested every six months during cell culture, and all results were negative.

Plasmids construction

Plasmids encoding mutant KRAS (G12V, G12D), p53 (R175H), HLA alleles (A0201, A0301, A1101, C0802), and immune molecules (CD80, CD8, PD-1) were constructed using their respective human full-length sequences as templates. The plasmid OKT3-scFv fusion protein links the signal peptide of CD5 and the transmembrane region of CD14 to OKT3-scFv. The reporter gene plasmid is designed to express the ZsGreen fluorescent protein, driven by the *IL-2* promoter, *NR4A1* promoter, and an engineered *8×NFAT:AP-1* promoter. Target DNAs were cloned into pSIN lentiviral vectors using the Hieff Clone Plus Single Step Cloning Kit (Yeasen).

For TCR plasmid construction, pSIN lentiviral vectors were modified by replacing the EF-1α promoter with the MND promoter to enhance expression efficiency. In order to distinguish the endogenous TCR, the constant regions of human TCR α chain as well as β chain were replaced with the constant regions of mouse TCR α chain as well as β chain, respectively. The key structure of the whole plasmid is as follows: MND promoter–human TCRα variable region–mouse TCRα constant region–P2A–human TCRβ variable region–mouse TCRβ constant region.

Flow cytometry and antibodies

Data were collected using a BD FACSCanto II flow cytometer and analysed using FlowJo software (version 10.0.7). Primary antibodies used for flow cytometry in this manuscript are listed below along with the supplier: anti-human CD3-APC antibody (BioLegend, clone OKT3), anti-human CD3-Pacific Blue antibody (BioLegend, clone UCHT1), anti-human CD8-APC antibody (BioLegend, clone RPA-T8), anti-human CD8-Pacific Blue antibody (BioLegend, clone RPA-T8), anti-mouse TCRβ-PE antibody (BioLegend, clone H57-597), anti-human HLA-A02-APC antibody (BioLegend, clone BB7.2), anti-human HLA-A03-FITC antibody (Thermofisher, clone GAP.A3), anti-human PD-1-Pacific Blue antibody (BioLegend, clone EH12.2H7), anti-human PD-L1-APC antibody (BioLegend, clone 29E.2A3), anti-human CD69-PE antibody (BioLegend, clone FN50), anti-human CD2-APC antibody (BioLegend, clone RPA-2.10), anti-human CD58 antibody (BioLegend, clone TS2/9), anti-mouse CD8-PerCP-Cy5.5 antibody (BioLegend, clone 53-5.8),

$OVA_{257-264}$ (SIINFEKL) peptide bound to H-2Kb-APC monoclonal antibody (Thermofisher, clone 25D1.16), anti-mouse IgG-FITC antibody (Beyotime).

Lentiviral packaging and titer quantification

Lentivirus packaging was carried out using the target lentiviral plasmid DNA, psPAX2, and pMD2.G in a 4:3:1 ratio. After 12 h of transfection, the medium was refreshed with culture medium containing 2 mM L-Glutamine, non-essential amino acids, and 1 mM sodium pyruvate (Gibco). At 48 h post-transfection, the virus-containing medium was collected and filtered through a 0.45 μm sterile syringe filter. For stable cell line construction, 1 mL of lentivirus and polybrene at a final concentration of 6 μg/mL were added to 1×10^5 cells.

To generate TCR-T cells, lentiviral stocks were concentrated and titrated. The lentivirus was concentrated 10-fold by centrifugation at 26,000 × g for 1.5 h at 4°C, removal of the supernatant, and resuspension in 1/10 of the original medium volume. For titration, a six-step, tenfold serial dilution was prepared, and each dilution was co-cultured with 1×10^5 Jurkat cells in a 24-well plate, with lentiviral transduction enhancer (LVTE, Vigene) added. After 24 h, the medium was replaced, and flow cytometry was performed at 72 h to detect mouse TCRβ expression on the Jurkat cell membrane. The lentiviral titer was calculated from wells exhibiting an expression rate of 10-30%, as follows: titre (TU/mL) = percentage of TCRβ positivity × number of cells / volume of lentiviral stock solution.

Western blot assay

Proteins were separated by SDS–PAGE using 10% Tris–glycine gels for 1 h and transferred onto PVDF membranes (0.45 μm, Millipore). Blocked the membrane with 5% skimmed milk in TBS-T buffer (20 mmol/L Tris–HCl, 150 mmol/L NaCl, 0.1% Tween 20, pH = 7.6) for 1 h. Ras G12V mutant specific antibody (Cell Signaling Technology, clone D2H12) and anti-GAPDH antibody (Diagbio, clone DGR11217) were diluted and co-cultured overnight at 4°C. The secondary antibody, goat anti-rabbit IgG (H+L)–HRP (Diagbio), was used at 1:5000 in 5% skim milk and incubated for 1 h at room temperature. Chemiluminescence was detected using Western Lightning Plus-ECL (Shanghai Pufei Biotechnology) and imaged with an Amersham Imager 680 (GE Healthcare).

RNA extraction and quantitative real-time PCR

Total RNA was extracted using RNAiso plus kit (TAKARA). A total of 2 μg of mRNA was then reverse-transcribed into cDNA using the cDNA synthesis kit (TransGen Biotech). Quantitative real-time PCR (qPCR) was performed using SYBR Green PCR Master Mix (Bio-Rad) on the qPCR platform (QuantStudio 6 Flex Real-Time PCR System, Thermofisher). Relative gene expression was normalized to β-actin. The specific primer pairs (5′→3′) for each gene are listed as follows: p53-F: CTTGCATTCTGGGACAGCCAAGTC; $p53^{R175H}$-specific-R: GAGCAGCGCTCATGGTGGGGGCAGT; WT-specific blocker-R: GCTCATGGTGGGGGCAGC-phospho; NY-ESO-1-F: TGCTTGAGTTCTACCTCGCCA; NY-ESO-1-R: TATGTTGCCGGACACAGTGAA; MAGE-A10-F: CACAGAGCAGCACTGAAGGAG; MAGE-A10-R: CTGGGTAAAGACTCACTGTCTGG; β-actin-F: CTGGAACGGTGAAGGTGACA; β-actin-R: CGGCCACATTGTGAACTTTG.

TCR-knockout Jurkat cells based on CRISPR-Cas9

CRISPR sgRNAs for TCR-knockout (KO) Jurkat cells were designed using http://crispor.tefor.net/. The annealed sgRNA were inserted into pSpCas9(BB)-2A-GFP (PX458) plasmid to generate PX458-TRAC and PX458-TRBC. The sgRNA sequences were TRAC-sgRNA: TGTGCTAGACATGAGGTCTATGG and TRBC-sgRNA: AGGCTTCTTCCCTGACCACGTGG. The plasmid pX458-TRAC was transfected into Jurkat cells using electroporation (Lonza). Jurkat cells expressing green fluorescent protein were then isolated by single-cell sorting using flow cytometry and cultured in 96-well plates. CD3-negative cells were identified via flow cytometry and subsequently electroporated with PX458-TRBC to achieve TCRβ chain KO. TCRβ KO cell lines were screened by reintroducing the TCRα chain, followed by CD3 detection via flow cytometry. Successful KO of both TCRα and TCRβ chains was confirmed by gene sequencing.

SignalFunc Index detection

The TCR-SignalFunc system was established by introducing a specific TCR and CD8 into TCR-knockout Jurkat cells. To enhance TCR expression, the MND promoter was employed. The system was co-cultured with target cells at a defined ratio for 12–24 h at 37 °C. Following co-culture, cells were harvested and the SignalFunc Index was determined by quantifying the percentage of

ZsGreen-positive cells within the $CD3^+$ Jurkat population using flow cytometry. For tissue-derived single-cell suspensions, the number of target cells was calculated based on their proportion within the multicellular mixture. For peptide-pulsing experiments, lyophilized peptides (Bankpeptide, 95% purity) were dissolved in DMSO to a concentration of 10 mM. Synthetic peptides used in this study included: HLA-A0201-restricted NY-ESO-$1_{157–165}$ (SLLMWITQC); HLA-A0201-restricted MAGE-$A10_{254-262}$ (GLYDGMEHL); HLA-A0201-restricted $p53^{R175H}$ (HMTEVVRHC); HLA-C0802-restricted $KRAS^{G12D}$ (GADGVGKSA 9mer); HLA-C0802-restricted $KRAS^{G12D}$ (GADGVGKSAL 10mer).

Construction of TCR-T cells

Primary human PBMCs were obtained from Shanghai Saily Biotechnology and transduced with TCR-encoding lentiviruses. One day before activation, untreated 24-well plates (BD Biosciences) were coated with 3 μg/mL anti-CD3 antibody (clone OKT3, BioLegend) in PBS and incubated overnight at 4 °C. The coating solution was removed, and 1×10^6 PBMCs per well were seeded in culture medium supplemented with 5 μg/mL anti-CD28 antibody (clone CD28.2, BioLegend), 10 ng/mL IL-7 (PeproTech), and 10 ng/mL IL-15 (PeproTech). After 48 h, T cell activation was assessed by flow cytometry, and the medium was replaced with concentrated lentiviral supernatant containing a lentiviral transduction enhancer (LVTE, Vigene) at a multiplicity of infection (MOI) of 5. Following 24 h of transduction, the supernatant was replaced with fresh medium containing 10 ng/mL IL-7, 10 ng/mL IL-15, and 50 μM 2-mercaptoethanol. At 96 h post-transduction, TCR expression on PBMCs was analyzed by flow cytometry using an anti-mouse TCRβ antibody.

Detection of cytokines secreted by TCR-T cells

Effector cells (TCR-T cells) were co-cultured with target cells at a specified ratio in 48-well plates using RPMI 1640 medium supplemented with 10% FBS (Gibco). The co-culture system was centrifuged at 400 g for 10 min, and the supernatant was collected for subsequent cytokine quantification.

Quantification of IL-2 and IFN-γ cytokines was performed by ELISA using the human IFN-γ uncoated ELISA kit (88-7316, Thermofisher) and the human IL-2 uncoated ELISA kit (88-7025, Thermofisher). ELISA plates were coated with the capture antibody overnight at 4°C and then

blocked with ELISA diluent at room temperature for 2 h. Samples or standards were subsequently incubated overnight at 4°C. Detection antibody, avidin-HRP or streptavidin-HRP, and TMB substrate solution were sequentially added, with 2 M H_2SO_4 used to stop the reaction. Absorbance was measured at 450 nm using a microplate reader (TECAN). Non-transduced PBMCs were co-cultured with target cells as a negative control to establish baseline cytokine levels. Normalized cytokine concentrations were determined by interpolation from a standard curve after subtracting baseline values.

Assessment of cell lysis using annexin V staining

The percentage of cell lysis was assessed using the Annexin V, FITC or 633 apoptosis detection kit (DOJINDO). Effector cells (TCR-T cells) were co-cultured with target cells at a specified ratio in 48-well plates using RPMI 1640 medium supplemented with 10% FBS (Gibco). After co-culture, target cells were collected by centrifugation and washed twice with PBS. A 10-fold diluted Annexin V binding solution was added to the cell pellet, followed by 5 μL of Annexin V, FITC or APC conjugate, and 5 μL of PI solution. The cells were incubated with the reagents for 15 min at room temperature in the dark and analyzed by flow cytometry. The total percentage of early (Annexin V^+/PI^-) and late (Annexin V^+/PI^+) apoptotic cells was defined as the total cell lysis rate. Background lysis from co-cultures with non-transduced PBMCs was subtracted to calculate normalized cell lysis percentage.

Assessment of cell lysis by LDH release assay

The fold change in LDH release was assessed using an LDH cytotoxicity assay kit (Beyotime). Effector cells (TCR-T cells) were co-cultured with target cells at a specified ratio in 96-well plates using phenol red-free RPMI 1640 medium supplemented with 2% FBS (Gibco). After centrifugation, 80 μL of supernatant was transferred to a new plate and mixed with 38 μL of the LDH working solution (prepared from a 1:1:1 mixture of enzyme solution, lactate solution, and INT solution). Following a period of incubation in the dark, absorbance was measured at 490 nm when obvious color change occurred. The fold change in LDH release was calculated for each TCR-T group as the LDH value divided by that of non-transduced PBMCs.

Efficacy of TCR-T cells *in vivo*

HPAC and HPAC-C08 cell lines (2×10^6 cells per mouse) were subcutaneously inoculated into the axillary region of NSG mice. On day 7 post-inoculation, the mice received a tail vein injection of either 4095-10 TCR-T cells (1.4×10^7 total cells per mouse, containing 32% TCR-positive cells, equivalent to 4.48×10^6 TCR^+ cells per mouse) or control T cells (non-transduced PBMCs, 1.4×10^7 cells per mouse). Starting on the day of T cell transfer, the mice received subcutaneous injections of recombinant human IL-2 (10000 IU/mL in a 100 μL volume) every 48 h. To evaluate immune reconstitution efficiency, blood samples were collected from the retro-orbital venous plexus 48 h after T cell injection, and the frequency of human $CD3^+$ ($hCD3^+$) cells was quantified by flow cytometry following red blood cell lysis. Tumor volume and mouse body weight were monitored regularly throughout the study, and all surviving mice were euthanized on day 22 after T cell transfer for final endpoint analysis.

Processing of CDX models

A375-CDX models were generated by axillary inoculation of A375 cells in nude mice. MC38-EGFP CDX models were established similarly in C57BL/6 mice. Tumor tissues from CDX models were dissociated into single cells using a commercial digestion solution (Absin, abs9522). Briefly, tissues were minced into 1–3 mm^3 fragments, digested in 10 volumes of digestion solution at 37°C for 30 min, and then filtered through a cell strainer (biosharp, 100 μm). After digestion was quenched, the cells were centrifuged at 400 g for 5 min, washed with PBS, and resuspended to obtain a single-cell suspension.

In the A375 CDX model, the tumor cell percentage was determined by quantifying HLA-A02 positive cells using flow cytometry. For the MC38-EGFP CDX model, viable cells were identified with Fixable Viability Stain 450 (BD Biosciences), and the proportion of EGFP positive cells was measured to determine the tumor cell percentage. Additionally, single-cell suspensions derived from the MC38-EGFP model were permeabilized using 0.25 μL/mL Triton X-100 and stained with a solution containing 0.05 mg/mL PI and 0.05 mg/mL RNase in PBS for 10 min, followed by flow cytometric analysis to assess DNA ploidy.

Processing of patient-derived samples

Peripheral blood was collected from patients, and PBMCs were isolated using a human lymphocyte separation medium (Dakewei). The presence of specific HLA types was initially assessed by flow cytometry, followed by high-resolution HLA typing sequencing to determine detailed HLA genotypes.

Patient-derived samples from surgically resected colorectal tumor tissues and adjacent non-tumorous tissues were dissociated into single cells using the same protocol as for CDX models, but with a reduced digestion time of 15 min. Detailed information on the patient-derived tissue samples is provided in **Table S2**, in accordance with the BRISQ reporting guidelines. Single-cell suspensions derived from patient-derived samples were permeabilized using 0.25 μL/mL Triton X-100 and stained with a solution containing 0.05 mg/mL PI and 0.05 mg/mL RNase in PBS for 10 min, followed by flow cytometric analysis to assess DNA ploidy.

Construction of TCR single-site saturated mutation library

The key structure of the entire JDI TCR wild-type plasmid is as follows: MND promoter–JDI TCRα variable region–mouse TCRα constant region–P2A–JDI TCRβ variable region–mouse TCRβ constant region. TCR single-site saturated mutation library was generated through site-saturated mutagenesis employing overlapping extension PCR, as previously detailed.[68] In summary, NNK-based mutagenesis was performed on the CDR1, CDR2, and CDR3 segments of the TCRα and TCRβ genes. The PCR products were subsequently inserted into the vector via homologous recombination and packaged into lentivirus. TCR library lentiviruses were used to transduce TCR-KO chassis cells stably expressing CD8 and an *8×NFAT:AP-1–ZsGreen* reporter at an MOI of 0.2.

Sample preparation for deep sequencing

The initial and sorted cell libraries were lysed, and total RNA was extracted using RNAiso plus kit (TaKaRa). Subsequently, the total RNA was reverse transcribed into cDNA using the protocol provided by the cDNA synthesis kit (TransGen Biotech). The resulting cDNA was used as the template for subsequent amplification processes. Primers for CDR library amplification (5′–3′) were as follows: CDR1α, F GCCCAGAAAGTGACCCAGGC; R CTGGCTGGCGGTGATGGTAAAG;

CDR2α, F GTGTACGAGACCAGAGACACCAC; R (shared with CDR1α) CTGGCTGGCGGTGATGGTAAAG; CDR3α, F CTGGAATTTCCAGAAGTCCACATCCTC; R CTTGCTATCCCGCAGCTGGTACAC; CDR1β, F CGGCGTGACCCAGACCCCTAAATTTC; R CAGGCCCAGCAGGAAGTTCTG; CDR2β, F GCCCAGGACATGAACCACGAG; R (shared with CDR1β) CAGGCCCAGCAGGAAGTTCTG; CDR3β, F GTCCCGGCTGAAAAAGCAGAAC; R GCCTTCTGGGTGTGGCTGATCTC. The PCRs were performed using high-fidelity DNA polymerase (TAKARA, R010A) in 50 μL reaction volumes, following the recommended cycling conditions. After quality control, the samples were subjected to deep sequencing on the Illumina MiSeq platform.

Statistical analysis

Statistical analyses were conducted using GraphPad Prism (version 8.0.1). Two-tailed unpaired Student's t-tests were used for comparisons between two groups, and one-way analysis of variance (ANOVA) followed by Dunnett's multiple comparisons test was used for multiple group comparisons. $P < 0.05$ was considered significant. ns, $P \geq 0.05$; $^{*}P < 0.05$; $^{**}P < 0.01$; $^{***}P < 0.001$.

Supplementary Figures

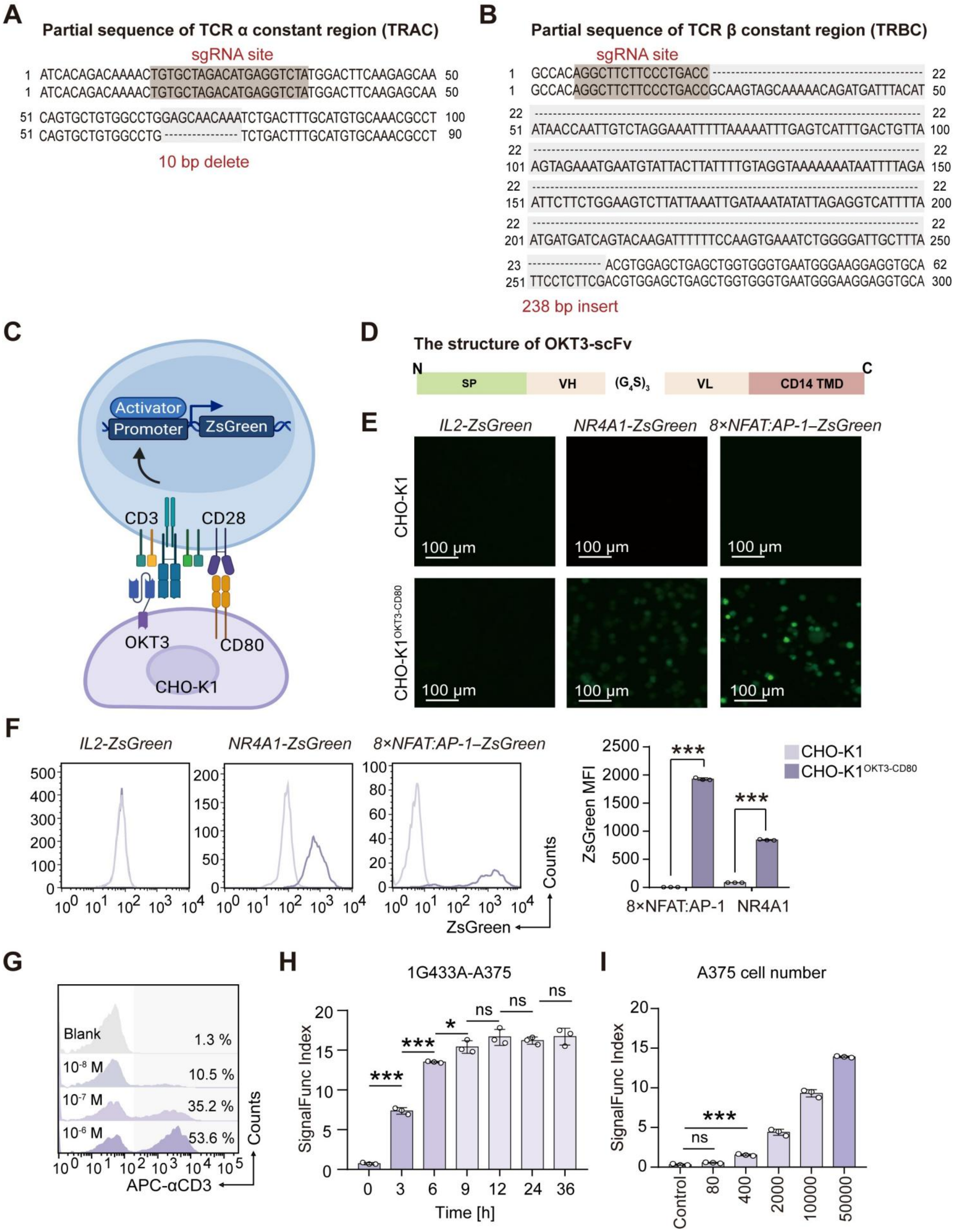

Fig. S1. Establishment of the TCR-SignalFunc system: chassis cell construction and SignalFunc Index definition. **(A-B)** Generation of TCR-knockout Jurkat cells using the CRISPR-Cas9 system. Sequencing of *TRAC* and *TRBC* genes to confirm successful gene knockout. **(C)** Schematic of promoter selection in the TCR-SignalFunc system. ZsGreen was fused downstream of promoters from different TCR signaling pathways and transduced into Jurkat cells. OKT3 and CD80-engineered CHO-K1 cells (CHO-K1$^{OKT3\text{-}CD80}$) were used to activate TCR signaling. **(D)** Schematic of cell membrane–anchored anti-CD3 antibody for the generation of CHO-K1$^{OKT3\text{-}CD80}$. The variable regions of anti-CD3 antibody (OKT3) were fused with the transmembrane domain (TMD) of CD14 and a signal peptide (SP). **(E-F)** Responsiveness of *NFAT:AP*-1 (repeat 8-times, 8×), *IL-2*, and *NR4A1* promoters to TCR signaling. Jurkat cells transduced with *promoter-ZsGreen* constructs were co-cultured with CHO-K1$^{OKT3\text{-}CD80}$ or CHO-K1 (E:T = 1:1, 24 h). Activated cells were visualized by fluorescence microscopy (green) **(E)**, and fluorescence intensity was quantified by flow cytometry **(F)**. **(G)** The readout of the TCR-SignalFunc system. The 1G433A-system was co-cultured with T2 cells in the presence of varying peptide concentrations (E:T = 1:1, 24 h). **(H)** Effect of co-culture duration on the potency of the TCR-SignalFunc system. 1G433A-system was co-cultured with A375 cells, and the SignalFunc Index was measured at multiple time points. **(I)** Impact of tumor cell number on assay potency and sensitivity. The 1G433A-system was co-cultured with varying quantities of A375 cells for 24 h.

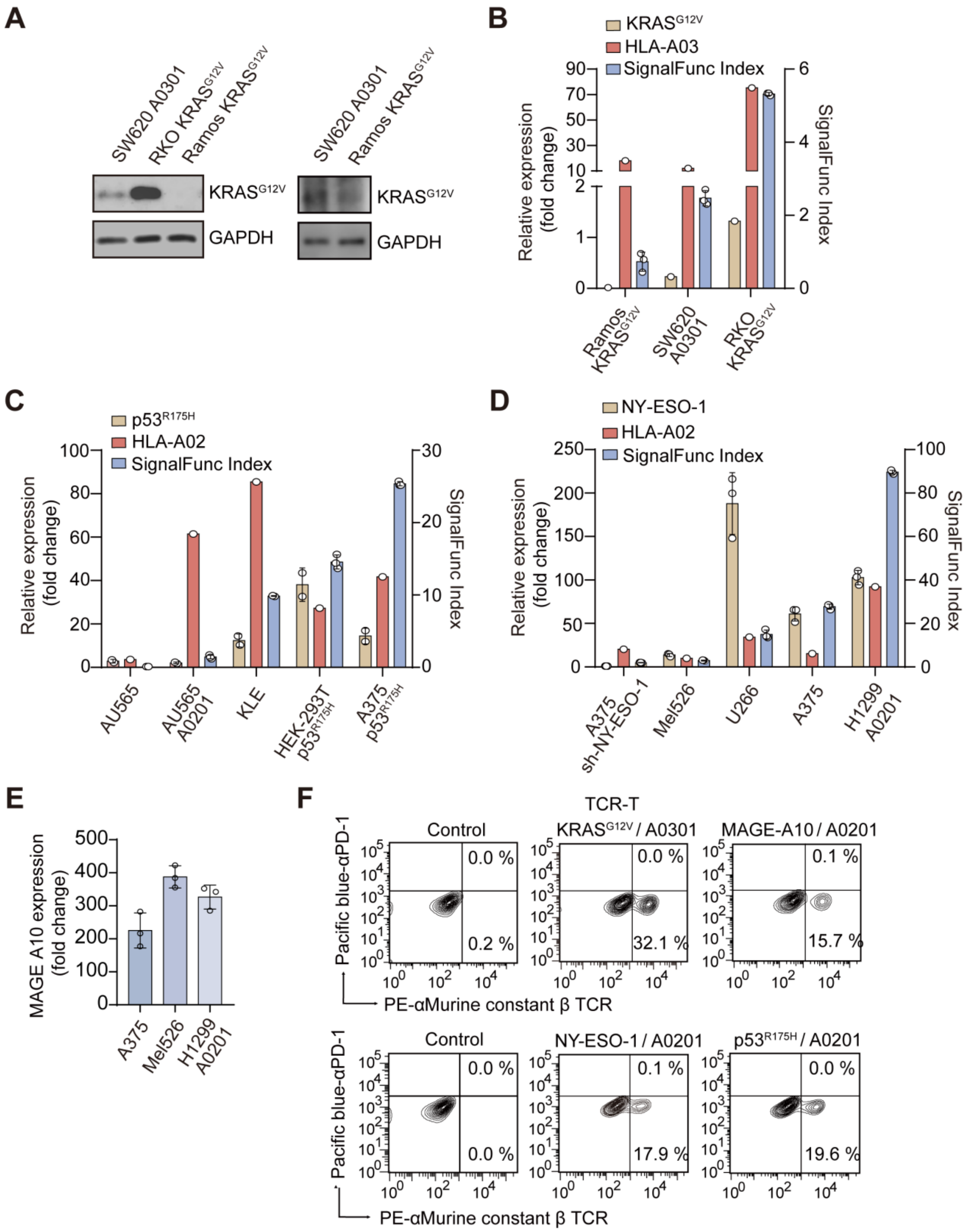

Fig. S2. Related gene expression of the cells used to demonstrate the correlation between SignalFunc Index of TCR-SignalFunc system and anti-tumor activity of TCR-T cells with the same TCRs. (A) Expression levels of KRAS^{G12V} in the relevant cell lines. KRAS^{G12V} expression was detected by western blot with mutant-specific antibodies, and the fold change relative to GAPDH was calculated. **(B)** Correlation between KRAS^{G12V}/HLA-A0301 neoantigen expression and SignalFunc Index. The SignalFunc Index was measured at a cell ratio of 1:1 for 24 h. **(C)** The correlation between p53^{R175H}/HLA-A0201 neoantigen expression levels and SignalFunc Index. Mutant-specific reverse primer (p53^{R175H}-specific-R), a competitive wild-type-specific reverse DNA oligo (WT-specific blocker-R), and WT cDNA-specific forward primer (p53-F) were used to amplify mutant-specific amplicons p53^{R175H} by quantitative real-time PCR (qPCR).

qPCR results were normalized to β-actin and expressed as fold changes relative to control samples (p53 wild-type H1299 cell line). The SignalFunc Index was measured at a cell ratio of 1:1 for 24 h. **(D)** The correlation between NY-ESO-$1_{157–165}$/HLA-A0201 tumor-associated antigen expression levels and SignalFunc Index. NY-ESO-1 mRNA levels were determined by qPCR, normalized to β-actin, and expressed as fold changes relative to control samples (A375 sh-NY-ESO-1 cell line). The SignalFunc Index was measured at a cell ratio of 1:1 for 24 h. **(E)** MAGE-A10 mRNA levels in cell lines were determined by qPCR, normalized to β-actin, and expressed as fold changes relative to a MAGE-A10-negative cell line. **(F)** The TCR or PD-1 expression levels of different TCR-T cells. The murine constant TCRβ and PD-1 expression in TCR-T cells were detected by flow cytometry. All replicates are shown as mean ± SD.

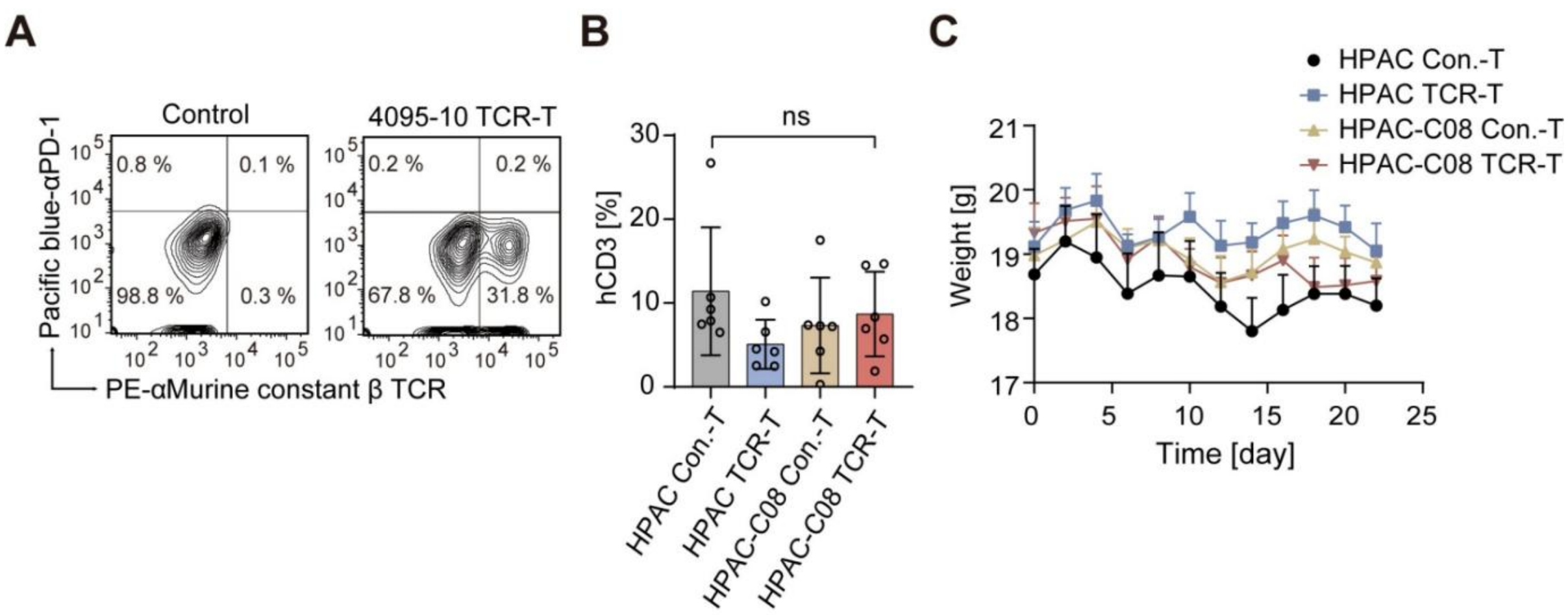

Fig. S3. Anti-tumor activity of 4095-10 TCR-T cells *in vivo* targeting KRAS^{G12D}/HLA-C0802. (A) Murine constant TCRβ and PD-1 expression on TCR-T cells determined by flow cytometry. **(B)** Assessment of immune reconstitution efficiency. 48 h after tail vein injection of TCR-T cells, blood was collected from the retro-orbital venous plexus, red blood cells lysed, and the proportion of human CD3-positive cells (hCD3) measured by flow cytometry to assess immune reconstitution in NSG mice. **(C)** Mouse body weight throughout the experiment. Data are presented as mean ± SD. (ns, $P \geq 0.05$; *$P < 0.05$; **$P < 0.01$; ***$P < 0.001$).

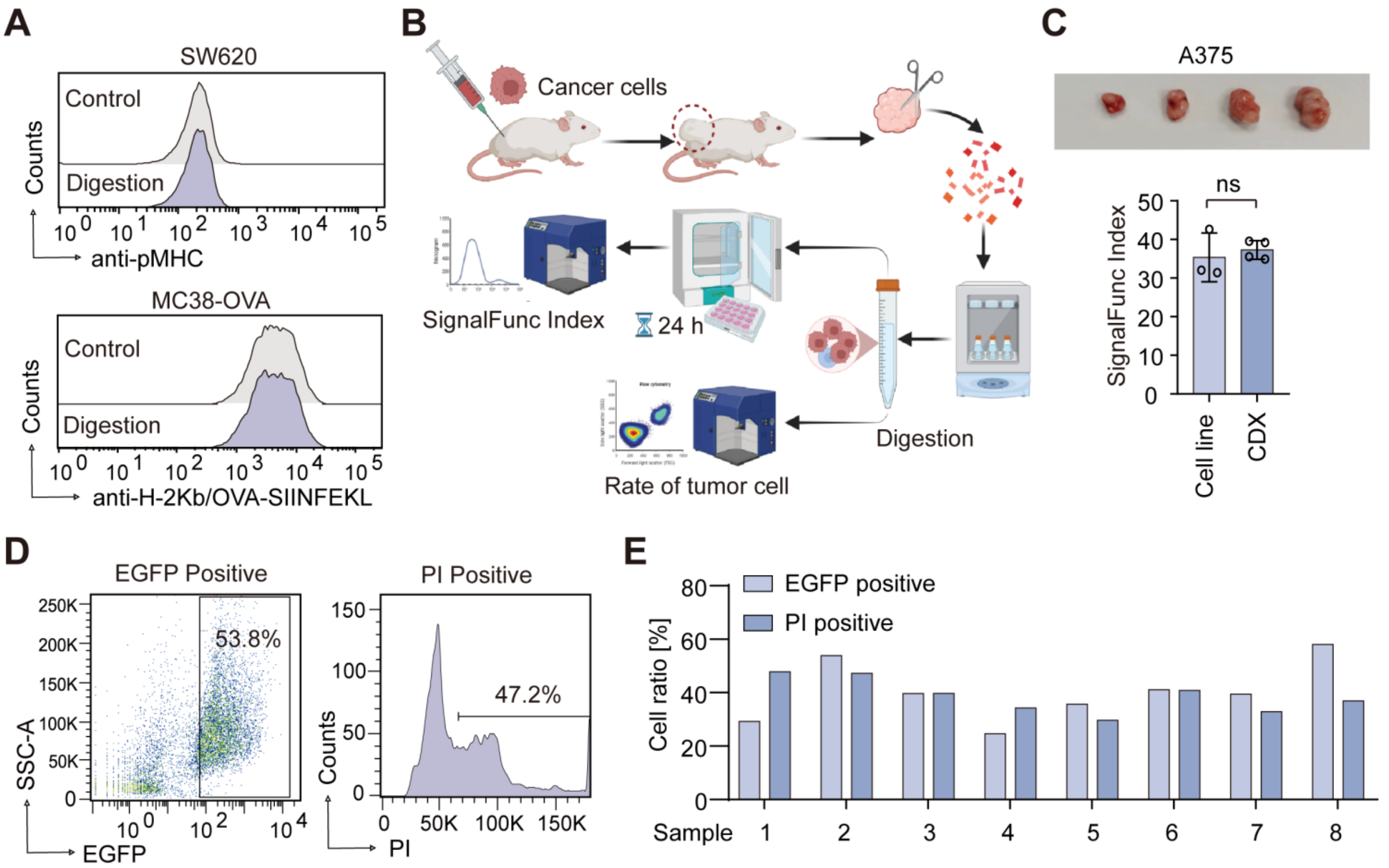

Fig. S4. Validation of the TCR-SignalFunc system for assessing TCR-T cell efficacy in tissue samples. (A) Effect of tissue digestion on cell-surface pMHC expression. SW620 and OVA-overexpressing MC38 cells were treated with tissue digestion solution for 30 min, and total pMHC (clone W6/32) or $H\text{-}2K^b$/OVA-SIINFEKL (clone 25-D1.16) levels were measured by flow cytometry. **(B)** Workflow of TCR-SignalFunc system based on tissue samples. Xenografts were generated, harvested, and digested into single-cell suspensions. Tumor cell proportion was determined by flow cytometry, then tumor cells were co-cultured with the TCR-SignalFunc system (E:T = 1:1, 24 h), and the SignalFunc Index was measured. **(C)** Comparison of SignalFunc Index induced by A375 cell line and its xenograft (CDX). Xenografts were processed into single cells, and HLA-A02 was detected by flow cytometry to quantify the proportion of tumor cells. Equal numbers of CDX-derived A375 cells and passaged A375 cells were co-cultured with the TCR-SignalFunc system specific for NY-ESO-1$_{157–165}$/HLA-A0201 (E:T = 1:1, 24 h). **(D–E)** Feasibility of assessing tumor cell proportion via DNA ploidy. MC38 cells stably transfected with EGFP served as a standard for proportion analysis. Aneuploid cells were defined as PI-positive. Single-cell suspensions were incubated for 10 min in PBS containing 0.25 μL/mL Triton X-100, 0.05 mg/mL RNase, and 0.05 mg/mL PI, followed by flow cytometric analysis; **(D)** showing the flow cytometry plot of a representative sample and **(E)** showing the comparison of tumor cell proportion analysis using EGFP and PI staining. All replicates are shown as mean ± SD. (ns, $P \geq 0.05$; *$P < 0.05$; **$P < 0.01$; ***$P < 0.001$)

A

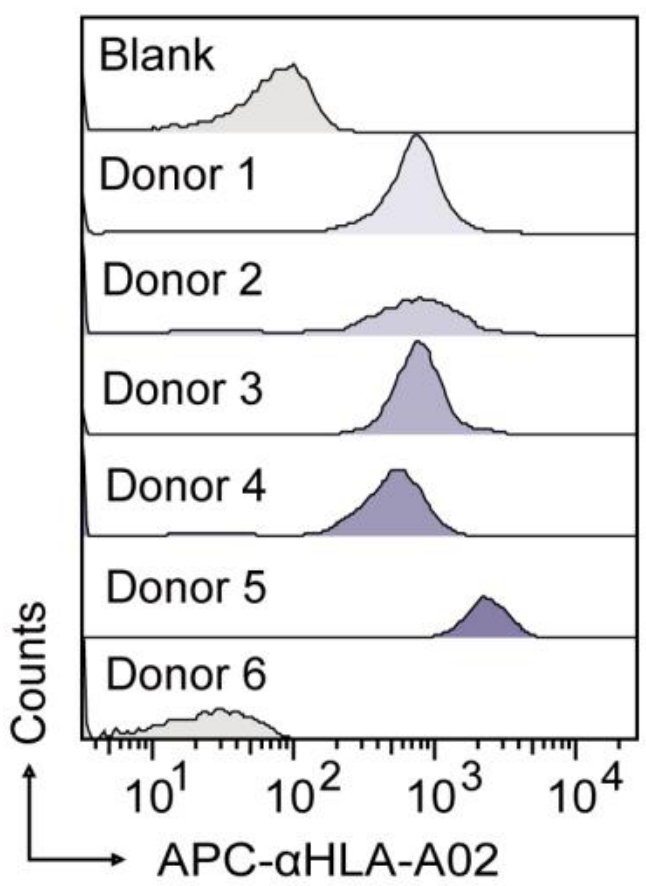

B

	HLA-A*	HLA-B*	HLA-C*
**Donor 1**	**02:01**, 11:01	15:01, 55:02	08:01, 12:03
**Donor 2**	**02:01**, 30:01	13:02, 51:02	06:02, 15:02
**Donor 3**	**02:03**, 26:01	35:01, 38:02	03:03, 07:02
**Donor 4**	**02:07**, 30:01	13:02, 15:01	04:01, 06:02
**Donor 5**	**02:07**, 03:01	27:05, 46:01	01:02, 02:02
**Donor 6**	29:02, 31:01	15:01, 45:01	03:03, 06:02

C

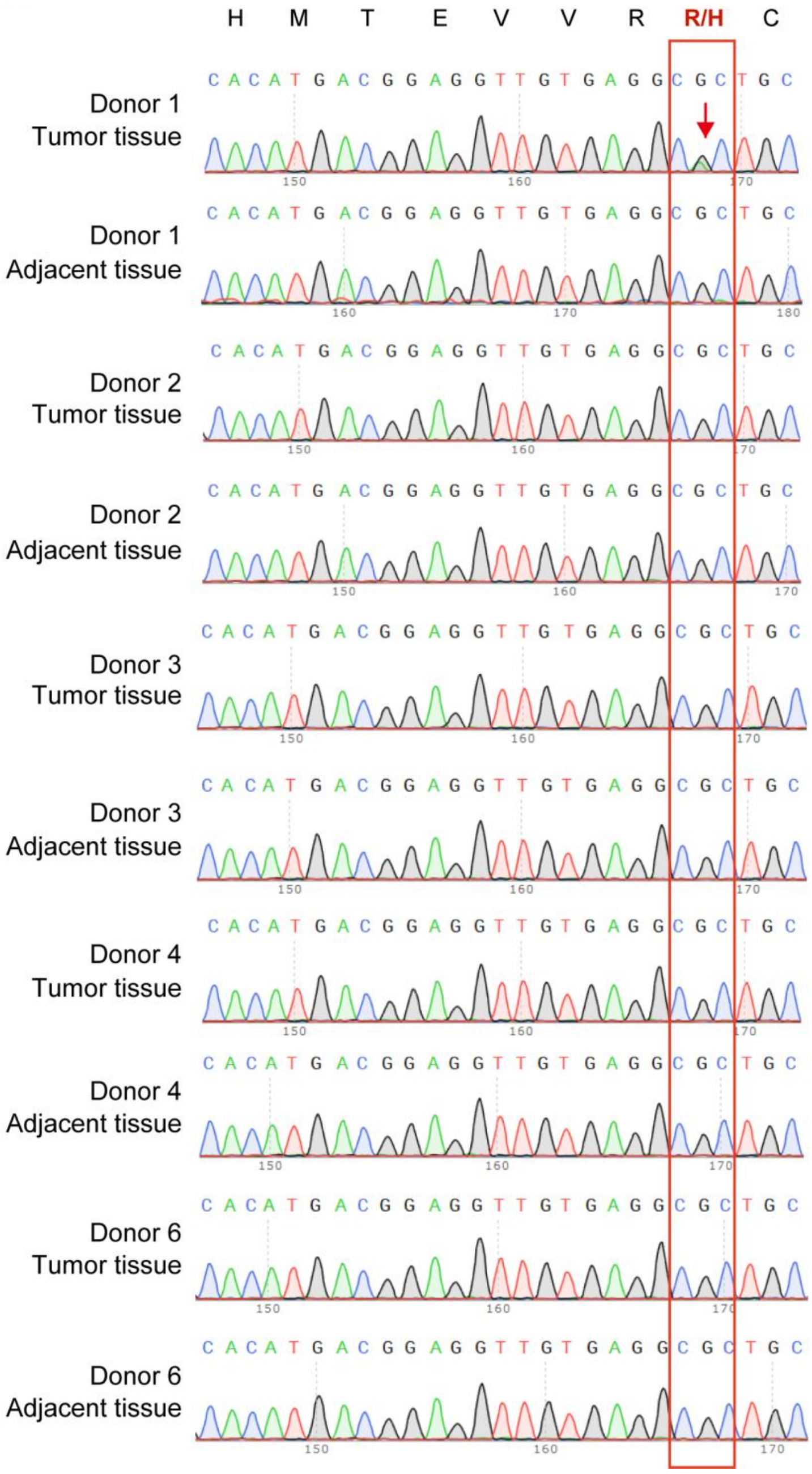

Fig. S5. HLA typing and p53 mutation status of patient donors. (A) Assessment of donor HLA-A02 expression by flow cytometry. PBMCs isolated from patient venous blood were analyzed to determine HLA-A02 surface expression. **(B)** High-resolution HLA typing via sequencing. Venous blood was collected from donors for high-resolution HLA sequencing to determine specific HLA alleles. **(C)** Mutation status of the p53 R175 locus in patient-derived tissue samples. DNA extracted from formalin-fixed paraffin-embedded (FFPE) tumor and adjacent normal tissues was amplified using specific primers and sequenced to identify the p53 R175 mutation.

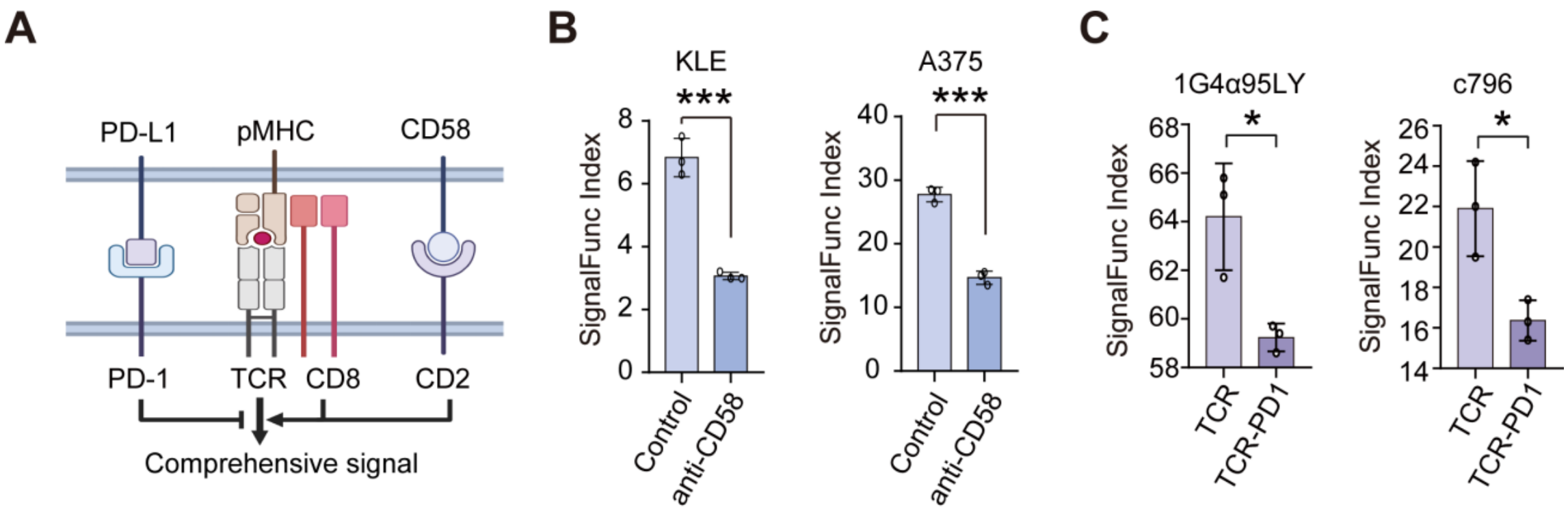

Fig. S6. Impact of co-stimulatory and co-inhibitory signaling on the SignalFunc Index. (A) Schematic of representative receptors for the modulation of TCR signaling. **(B)** CD2 costimulatory signal modulates the SignalFunc Index. 4196-AV12/BV6-system (targeting $p53^{R175H}$/HLA-A0201) and 1G4α95LY-system (targeting NY-ESO-$1_{157–165}$/HLA-A0201) were co-cultured with KLE ($p53^{R175H+}$/HLA-$A0201^{+}$) and A375 cells, respectively, in the presence of 10 µg/mL anti-CD58 antibody (E:T = 1:1, 24 h). **(C)** PD-1 immune checkpoint signal modulates the SignalFunc Index. PD1-transduced and non-transduced 1G4α95LY-system and c796-system (c796 TCR targeting MAGE-$A10_{254-262}$/HLA-A0201) were co-cultured with MDA-MB-231 cells (PD-$L1^{+}$) and 10^{-7} M peptide (E:T = 1:1, 24 h), respectively. All replicates are shown as mean ± SD (n = 3). (ns, $P \geq 0.05$; *$P < 0.05$; **$P < 0.01$; ***$P < 0.001$)

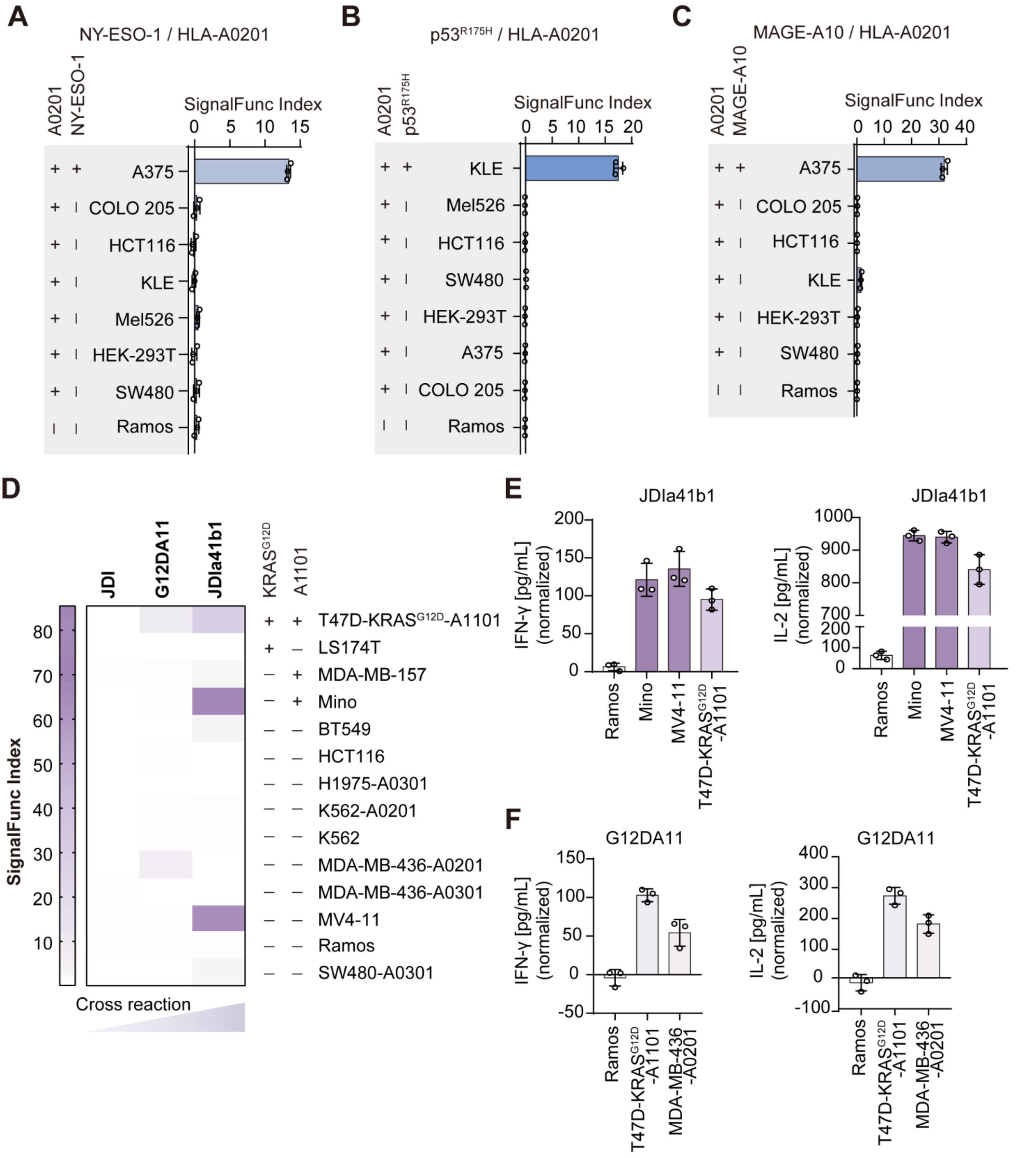

Fig. S7. SignalFunc Index is capable of indicating the cross-reactivity of TCRs. (A–C) Cross-reactivity of clinically tested TCRs. TCR-SignalFunc systems were co-cultured with antigen-positive or antigen-negative cells (E:T = 1:1, 24 h). 1G4α95LY TCR **(A)** is specific for NY-ESO-1$_{157–165}$/HLA-A0201. 4196-AV12/BV6 TCR **(B)** is specific for p53^{R175H}/HLA-A0201. c796 TCR **(C)** is specific for MAGE-A10$_{254-262}$/HLA-A0201. **(D)** Cross-reactivity of TCRs targeting KRAS^{G12D}/HLA-A1101. TCRs with significant differences in affinity are used to generate the TCR-SignalFunc systems, which were co-cultured with an antigen-positive cell line or a panel of antigen-negative cell lines (E:T = 1:1, 24 h). **(E–F)** Validation of TCR-SignalFunc system–identified cross-reactivity by assessing TCR-T cell activation. TCR-T cells transduced with JDIa41b1 TCR or G12DA11 TCR were co-cultured with target cells (E:T = 10:1, 14 h). IFN-γ and IL-2 levels in supernatants

were measured by ELISA and normalized to the control group after background subtraction. All replicates are shown as mean ± SD (n = 3).

A

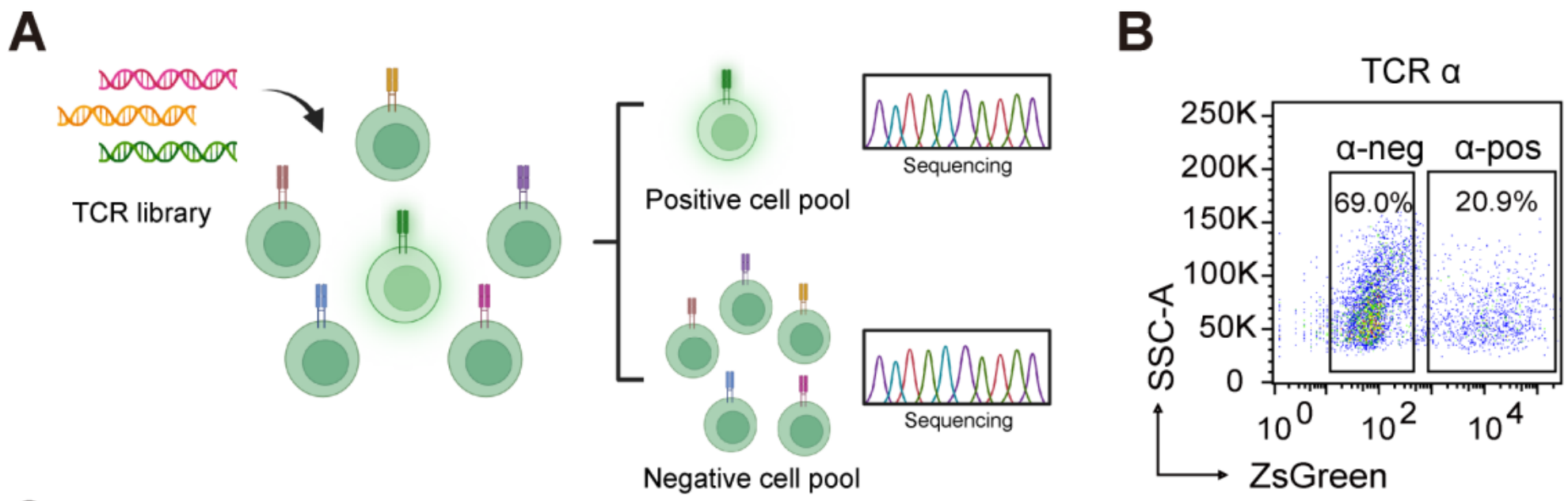

C

Ranking of enrichment ratios (α)

TCRα	Site	Mutation	Enrichment ratios
CDR1	P4	H	2.90
CDR1	P4	N	2.62
CDR1	P4	A	2.42
CDR1	P4	W	2.08
CDR1	P2	C	1.85
CDR2	P6	A	11.32
CDR2	P5	F	4.55
CDR2	P1	L	4.01
CDR2	P6	G	3.70
CDR2	P6	T	2.94
CDR3	P11	H	5.57
CDR3	P14	M	3.99
CDR3	P8	T	3.39
CDR3	P8	M	3.14
CDR3	P8	E	3.01

D

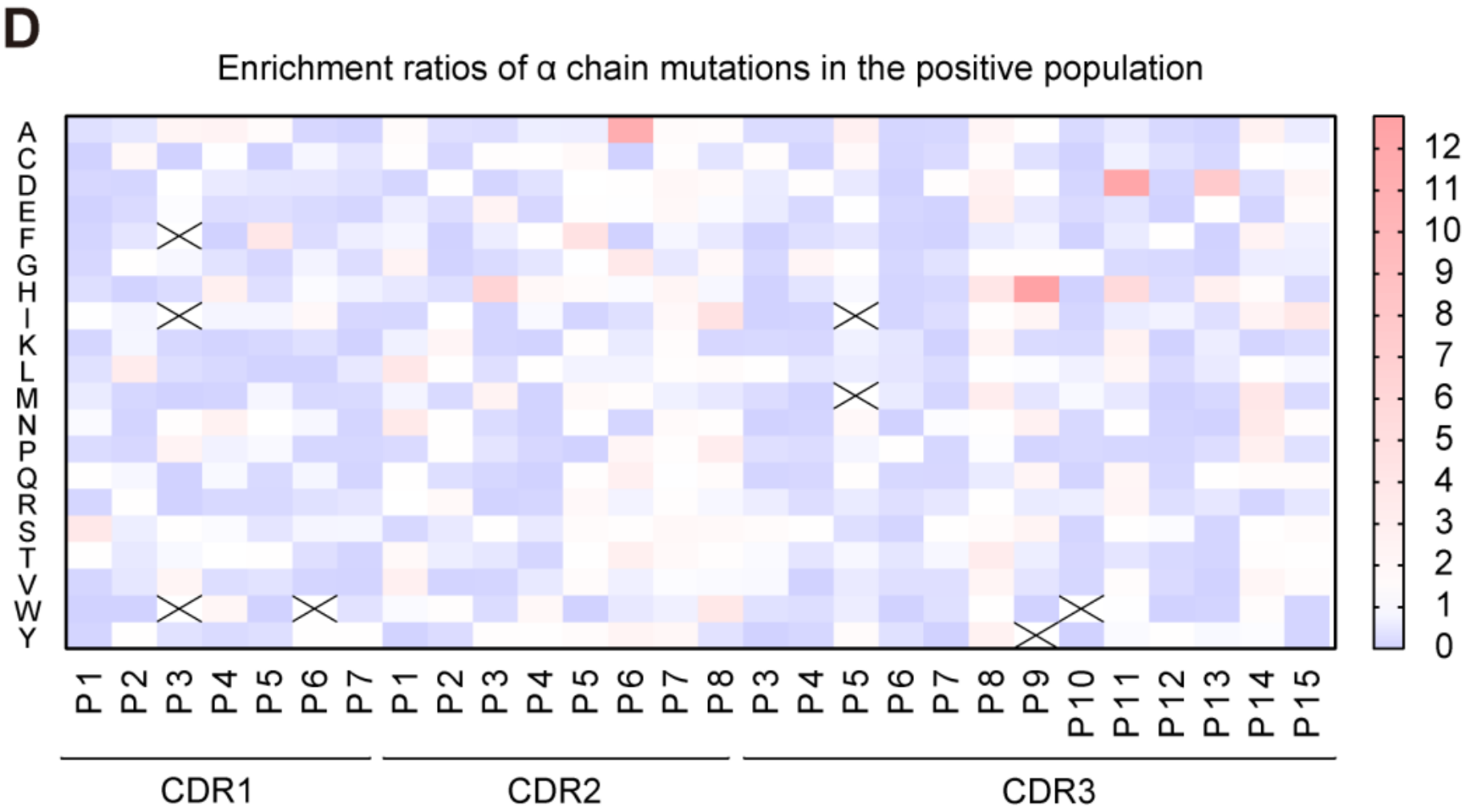

E

Ranking of enrichment ratios (β)

TCRβ	Site	Mutation	Enrichment ratios
CDR1	P5	M	3.10
CDR1	P5	L	3.07
CDR1	P4	D	2.95
CDR1	P5	D	2.82
CDR1	P2	V	2.46
CDR2	P4	Y	9.13
CDR2	P3	N	6.60
CDR2	P3	K	3.09
CDR2	P3	V	2.79
CDR2	P2	L	2.76
CDR3	P11	R	1.72
CDR3	P14	A	1.29
CDR3	P5	V	1.14
CDR3	P5	Y	1.02
CDR3	P6	G	1.02

F

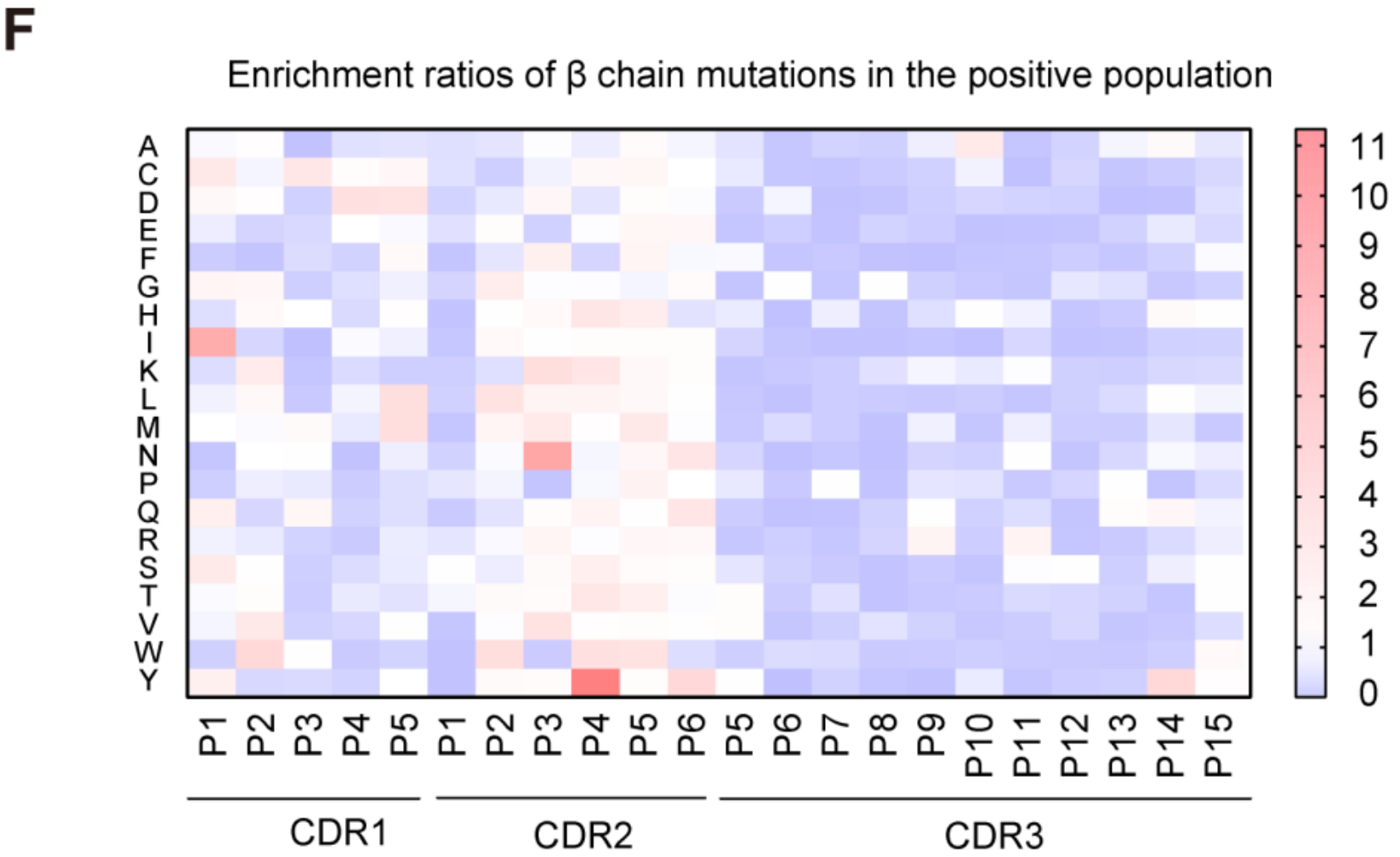

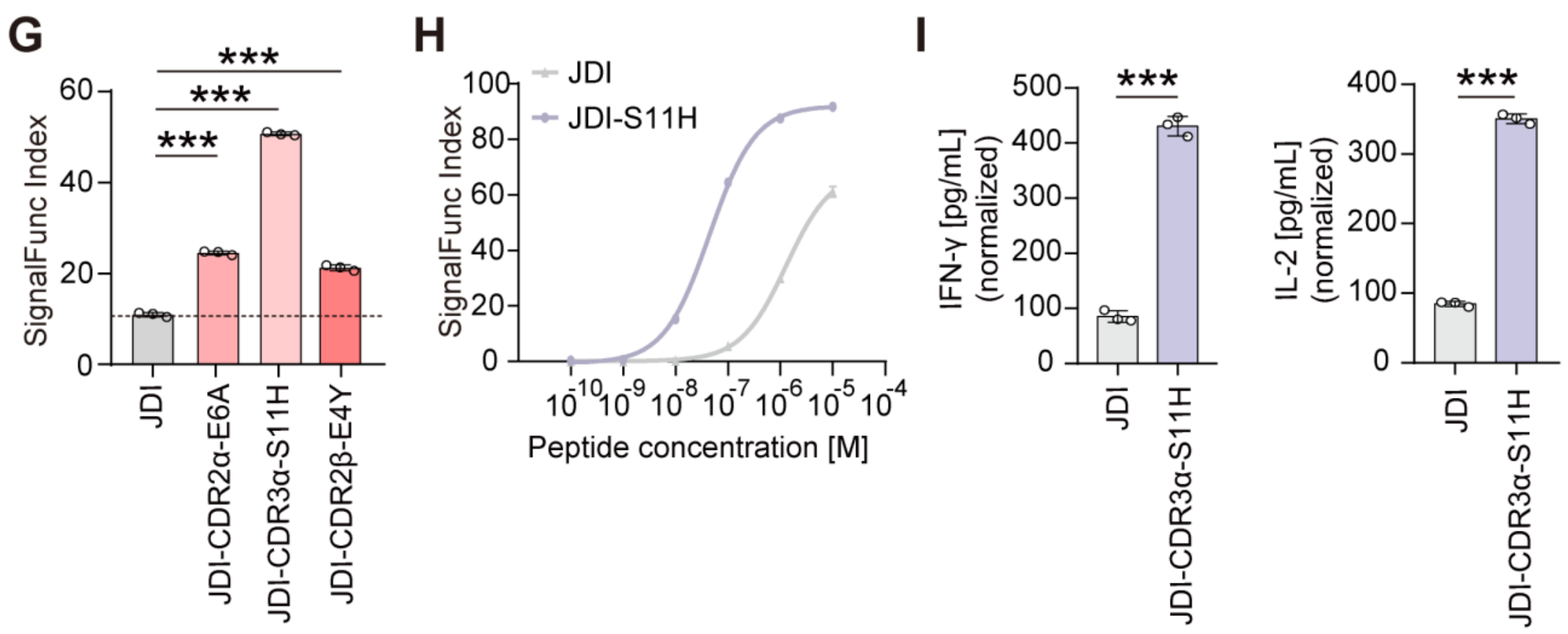

Fig. S8. Optimizing the therapeutic potential of JDI TCR targeting KRAS^{G12D}/HLA-A1101 via the TCR-SignalFunc system. (A–B) Workflow for high-throughput optimization of TCR therapeutic potential. Single-point saturation mutation library was constructed based on the CDR1, CDR2, and CDR3 sequences of the JDI TCR α and β chains **(A)**. The TCR-SignalFunc system based JDI TCR library was co-cultured with K562-A1101 cells at a ratio of 1:2 supplemented with 10^{-5} M peptide for 12 h, followed by sorting of SignalFunc Index-positive and -negative populations via flow cytometry **(B)**. Total RNA was extracted and reverse-transcribed into cDNA, after which primer-specific PCR was performed to obtain sufficient DNA for deep sequencing. **(C–F)** Enrichment ratios of mutant JDI TCR α and β chains in the SignalFunc Index-positive population (enrichment ratio = mutation frequency in positive population / that in initial library; mutations with enrichment ratio > 1 in the negative population excluded), with the top five enriched mutations in CDR1, CDR2, or CDR3 of TCR α **(C)** and TCR β **(E)**, and heatmaps of mutation enrichment for TCR α **(D)** and TCR β **(F)**. **(G)** Construction of mutant JDI TCR-systems to assess functional impact of mutations. These systems were co-cultured with K562-A1101 cells with 10^{-7} M peptide (E:T = 1:1, 24 h). **(H–I)** Comparison of the therapeutic potentials of JDI and JDI-CDR3α-S11H. TCR-SignalFunc systems transduced with these TCRs were co-cultured with K562-A1101 cells in the presence of peptide at various concentrations (E:T = 1:1, 24 h) **(H)**. TCR-T cells transduced with these TCRs were co-cultured with K562-A1101 cells in the presence of 10^{-5} M peptide (E:T = 2:1, 14 h); IFN-γ and IL-2 levels in supernatants were quantified by ELISA and normalized to the control group after background subtraction **(I)**. All replicates are shown as mean ± SD (n = 3) (ns, $P \geq 0.05$; *$P < 0.05$; **$P < 0.01$; ***$P < 0.001$).

Supplementary Tables

Table S1. List of TCRs used in the article.

TCR ID	Antigen source	Epitope sequence (N→C)	Epitope position	HLA restriction
1G4	NY-ESO-1	SLLMWITQC	157-165	HLA-A0201
1G4α95LY	NY-ESO-1	SLLMWITQC	157-165	HLA-A0201
1G433A	NY-ESO-1	SLLMWITQC	157-165	HLA-A0201
4095-10	KRAS G12D	GADGVGKSAL	10-19	HLA-C0802
4095A	KRAS G12D	GADGVGKSA	10-18	HLA-C0802
TCRA3V	KRAS G12V	VVVGAVGVGK	7-16	HLA-A0301
JDI	KRAS G12D	VVVGADGVGK	7-16	HLA-A1101
G12DA11	KRAS G12D	VVVGADGVGK	7-16	HLA-A1101
JDIa41b1	KRAS G12D	VVVGADGVGK	7-16	HLA-A1101
4196-AV12/BV6	p53 R175H	HMTEVVRHC	168-176	HLA-A0201
c796	MAGE-A10	GLYDGMEHL	254-262	HLA-A0201
CEA TCR	CEA	IMIGVLVGV	691-699	HLA-A0201
JDI-CDR2α-E6A	KRAS G12D	VVVGADGVGK	7-16	HLA-A1101
JDI-CDR3α-S11H	KRAS G12D	VVVGADGVGK	7-16	HLA-A1101
JDI-CDR2β-E4Y	KRAS G12D	VVVGADGVGK	7-16	HLA-A1101

Table S2. Detailed patient-derived tissue sample information according to BRISQ guidelines.

Category	Item (BRISQ Tier)	Information
Basic Sample Identification	Sample type (Tier 1)	Tumor tissue, adjacent non-tumorous tissue, peripheral blood
	Anatomical site / organ (Tier 1)	Colorectal region
	Disease status / diagnosis (Tier 1)	Pathologically confirmed colorectal cancer
Donor/Patient Information	Demographics (Tier 2)	Donor 1, male<br>Donor 2, female<br>Donor 3, male<br>Donor 4, male<br>Donor 5, male<br>Donor 6, male
Collection Details	Date/time of collection (Tier 1)	Donor 1, 2024-12-10 format<br>Donor 2, 2024-12-24 format<br>Donor 3, 2024-11-29 format<br>Donor 4, 2024-11-29 format<br>Donor 5, 2024-12-27 format<br>Donor 6, 2024-11-05 format
Processing & Preservation	Processing steps (Tier 1/Tier 2)	Tissues were dissociated into single cells using a commercial digestion solution. Briefly, tissues were minced into 1–3 mm^3 fragments, digested in 10 volumes of digestion solution at 37°C for 15 minutes, and then filtered through a cell strainer (biosharp, 100 μm). After digestion was quenched, the cells were centrifuged at 400 g for 5 minutes, washed with PBS, and resuspended to obtain a single-cell suspension.
Storage & Transport	Storage conditions (Tier 1)	Immediate processing
